# Supplementary material for: Transfer learning improves resting-state functional connectivity pattern analysis using convolutional neural networks
Source: Gigascience. 2018 Nov 5;7(12):giy130. doi: 10.1093/gigascience/giy130 (PMC6283213; doi:10.1093/gigascience/giy130)

GigaScience

Transfer learning improves resting-state functional connectivity pattern analysis using convolutional neural networks

--Manuscript Draft--

**Manuscript Number:**	GIGA-D-18-00100
**Full Title:**	Transfer learning improves resting-state functional connectivity pattern analysis using convolutional neural networks
**Article Type:**	Research
**Funding Information:**	Hungarian Brain Research Program (KTIA_13_NAP–A–I/18) — Dr. Zoltán Vidnyánszky
**Abstract:**	Background<br>Deep learning is gaining importance in the prediction of cognitive states and brain pathology based on neuroimaging data. Including multiple hidden layers in artificial neural networks enables unprecedented predictive power; however, the proper training of deep neural networks requires many thousands of exemplars. Collecting this amount of data is not feasible in typical neuroimaging experiments. A handy solution to this problem, which has largely fallen outside the scope of deep learning applications in neuroimaging, is to repurpose deep networks that have already been trained on large datasets by fine-tuning them to target datasets/tasks with fewer exemplars. Here we investigated how this method, called transfer learning, can aid age category classification and regression based on brain functional connectivity patterns derived from resting-state functional magnetic resonance imaging. We trained a connectome-convolutional neural network on a larger, publicly available dataset and then examined how the knowledge learned can be used effectively to perform these tasks on a smaller dataset collected in our lab with a different type of scanner, imaging protocol and pre-processing pipeline.<br>Results<br>Age classification on the in-house dataset benefitted from transfer learning. The most pronounced improvement was observed when the convolutional layers' weights were initialized based on the values learned on the public dataset and then fine-tuned to the in-house dataset. Transfer learning also brought significant improvement to the otherwise poor prediction of chronological age.<br>Conclusions<br>Transfer learning is a plausible solution to adapt convolutional neural networks to neuroimaging data with different data acquisition and pre-processing protocols.
**Corresponding Author:**	Pál Vakli, Ph.D.<br><br>HUNGARY
**Corresponding Author Secondary Information:**	
**Corresponding Author's Institution:**	
**Corresponding Author's Secondary Institution:**	
**First Author:**	Pál Vakli, Ph.D.
**First Author Secondary Information:**	
**Order of Authors:**	Pál Vakli, Ph.D.<br>Regina Júlia Deák-Meszlényi<br>Petra Hermann, Ph.D.<br>Zoltán Vidnyánszky, D.Sc.
**Order of Authors Secondary Information:**	
**Additional Information:**	

Powered by Editorial Manager® and ProduXion Manager® from Aries Systems Corporation

Question	Response
Are you submitting this manuscript to a special series or article collection?	No
**Experimental design and statistics**<br><br>Full details of the experimental design and statistical methods used should be given in the Methods section, as detailed in our Minimum Standards Reporting Checklist. Information essential to interpreting the data presented should be made available in the figure legends.<br><br>Have you included all the information requested in your manuscript?	Yes
**Resources**<br><br>A description of all resources used, including antibodies, cell lines, animals and software tools, with enough information to allow them to be uniquely identified, should be included in the Methods section. Authors are strongly encouraged to cite Research Resource Identifiers (RRIDs) for antibodies, model organisms and tools, where possible.<br><br>Have you included the information requested as detailed in our Minimum Standards Reporting Checklist?	Yes
**Availability of data and materials**<br><br>All datasets and code on which the conclusions of the paper rely must be either included in your submission or deposited in publicly available repositories (where available and ethically appropriate), referencing such data using a unique identifier in the references and in the "Availability of Data and Materials" section of your manuscript.<br><br>Have you have met the above requirement as detailed in our Minimum Standards Reporting Checklist?	Yes

Manuscript
Click here to download Manuscript Transfer_learning_manuscript.pdf

Transfer learning improves resting-state functional connectivity pattern analysis using convolutional neural networks

Pál Vakli[1*†], Regina J. Deák-Meszlényi[1,2*], Petra Hermann[1], Zoltán Vidnyánszky[1,2]

[1]Brain Imaging Centre, Research Centre for Natural Sciences, Hungarian Academy of Sciences, Magyar tudósok körútja 2., 1117 Budapest, Hungary

[2]Department of Cognitive Science, Budapest University of Technology and Economics, Egry József utca 1., 1111 Budapest, Hungary

*Pál Vakli and Regina J. Deák-Meszlényi contributed equally to this paper.

†Correspondence: Pál Vakli, Brain Imaging Centre, Research Centre for Natural Sciences, Hungarian Academy of Sciences, Magyar tudósok körútja 2., 1117 Budapest, Hungary. Tel: +361 3826446; E-mail: vakli.pal@ttk.mta.hu

E-mail addresses: vakli.pal@ttk.mta.hu (Pál Vakli), meszlenyi.regina@ttk.mta.hu (Regina J. Deák-Meszlényi), hermann.petra@ttk.mta.hu (Petra Hermann), vidnyanszky.zoltan@ttk.mta.hu (Zoltán Vidnyánszky)

Abstract

Background

Deep learning is gaining importance in the prediction of cognitive states and brain pathology based on neuroimaging data. Including multiple hidden layers in artificial neural networks enables unprecedented predictive power; however, the proper training of deep neural networks requires many thousands of exemplars. Collecting this amount of data is not feasible in typical neuroimaging experiments. A handy solution to this problem, which has largely fallen outside the scope of deep learning applications in neuroimaging, is to repurpose deep networks that have already been trained on large datasets by fine-tuning them to target datasets/tasks with fewer exemplars. Here we investigated how this method, called transfer learning, can aid age category classification and regression based on brain functional connectivity patterns derived from resting-state functional magnetic resonance imaging. We trained a connectome-convolutional neural network on a larger, publicly available dataset and then examined how the knowledge learned can be used effectively to perform these tasks on a smaller dataset collected in our lab with a different type of scanner, imaging protocol and pre-processing pipeline.

Results

Age classification on the in-house dataset benefitted from transfer learning. The most pronounced improvement was observed when the convolutional layers' weights were initialized based on the values learned on the public dataset and then fine-tuned to the in-house dataset. Transfer learning also brought significant improvement to the otherwise poor prediction of chronological age.

Conclusions

Transfer learning is a plausible solution to adapt convolutional neural networks to neuroimaging data with different data acquisition and pre-processing protocols.

Keywords

deep learning, transfer learning, convolutional neural networks, resting-state fMRI, brain age prediction

Background

Deep learning, a branch of machine learning that allows multi-layered neural network models to learn representing data at increasing levels of abstraction [1], is gaining importance in the analysis of brain imaging data [2], and has been applied successfully in neuroimaging studies of psychiatric and neurological disorders [3]. As an example, our group has successfully applied deep learning for fMRI-based classification of amnestic mild cognitive impairment [4]. More specifically, we presented a novel convolutional neural network (CNN) architecture that efficiently distinguished between subject groups based on functional connectivity metrics derived from resting-state fMRI measurements.

While these methods have the potential to revolutionize fMRI data analysis [2] and provide a conceptual framework for understanding brain function [5], training deep neural networks comes at a cost. This is mainly because many examples are required to properly train these models. A rough rule of thumb is that to achieve agreeable performance, a supervised deep learning algorithm requires around 5000 labelled training examples per category [6]. Accordingly, datasets used in several areas of machine learning are often enormous. For example, the AlexNet [7], a CNN model that achieved a breakthrough in natural image

recognition in 2012 [8], was trained on roughly 1.2 million examples from the ImageNet database [9]. This is in stark contrast with the sample size in typical neuroimaging experiments. In a recent review of more than 200 studies using neuroimaging and machine learning for the classification of patients with various brain disorders, the authors found that the median sample size of all studies was 88 [10]. By contrast, the number of features (regions or voxels) in neuroimaging experiments is typically far greater—in the field of functional connectomics, it ranges from the order of tens to 1 million [11]. Complex models trained under such circumstances are prone to learn the idiosyncratic details of the sample data instead of the general functional relationship between brain activation patterns and cognitive states. For this reason, such models show poor generalization to samples they have never encountered before, a phenomenon which is commonly referred to as 'overfitting' [12,13].

Open sharing of neuroimaging data is envisaged by many as a possible solution to the problem of small sample sizes [10]. Significant progress has been made in this area, as now there are more than 8000 shared MRI datasets available online [14]. However, data sharing entails the possibility of introducing undesirable variability into data analysis, which is a central issue in multicenter fMRI studies, and is related to differences in scanner types, sequence parameters, stimulus presentation, and image processing between research sites [15]. In addition, the increased computational burden of processing vast amounts of neuroimaging data should also be taken into account [2]. Considering these limitations, the question arises as to how data from different sources can be combined effectively for deep learning applications in neuroimaging.

In machine learning, it is not uncommon to rely on previous knowledge instead of training a model from scratch. Transfer learning [16] refers to the method of training a model on one dataset (the source domain) and then transfer the acquired knowledge—which is, in the case of neural networks, manifest in the learned weights—to train a model on a different dataset and/or task (the target domain). This method is useful when the source and target datasets differ in

terms of feature space or data distribution [16] and can be used effectively when the target dataset is too small to train a large network without overfitting [17]. As a recent example, [18] harnessed the image representations learned by a CNN on a large-scale dataset (the ImageNet; see above) in order to perform various visual recognition tasks on a dataset with only limited amount of training examples. In particular, the pre-trained parameters of the internal layers were transferred to the target task and kept constant, while the last fully connected layer was replaced by two new layers that were trained on the target dataset. This transfer learning method led to enhanced performance when compared to state-of-the-art models, despite differences in image statistics and tasks between the two datasets [18]. Other examples include keeping the weights of the pre-trained layers fixed and training a linear regression or support vector machine (SVM) classifier on top to adapt the model to the target domain [19–21].

[17] trained a CNN for visual classification on one dataset and then systematically examined the extent to which transferring parameters from different layers aids the retraining of the remaining layers on a similar dataset. The authors found that the first two layers show almost perfect transfer, in line with the frequently observed phenomenon that when deep neural networks are trained on images, the resulting representations in their first layers—i.e. Gabor filters or color blobs [22]—are general in the sense that they can be applied to many datasets and tasks. Transferring deeper layers, however, led to a significant drop in performance due to the representations being more specific to the source domain as well as due to the loss of co-adapted representations between successive layers. Interestingly, transferring the weights only to initialize the network which is then fine-tuned to the target dataset resulted in better performance than when the network was trained directly on the target dataset. This suggests that transfer learning may be desirable even when the target domain has sufficient examples to train the network without overfitting [17].

Taken together, the above results suggest that transfer learning is beneficial when the sample size in the target domain is too small to train deep neural networks without overfitting. The effectiveness of this method depends on the use of knowledge about the source domain, i.e. which layers are transferred and whether the weights are fixed or used only to initialize the network when training on the target dataset. While these studies focused on how to deal with the scarcity of data in specific natural image recognition tasks, transfer learning has to potential to alleviate the problem of small sample size in neuroimaging.

In the present study, we performed a systematic investigation of how knowledge can be extracted effectively from a model that has already been trained on a publicly available dataset. In particular, we examined how transfer learning can be used to adapt a CNN to a relatively small dataset to predict age from functional neuroimaging data. Predicted brain age is attracting significant attention due to its potential as a biomarker of individual brain health [23], and recent results show that deep learning is effective in predicting age from structural MRI data [24]. In the current study, region-of-interest-based whole-brain resting-state functional connectivity matrices acquired in our own lab from subjects of two age categories (elderly and young) constituted the target domain. The source domain consisted of functional connectivity matrices and corresponding chronological age labels from a publicly available dataset. The two datasets differed markedly in size and data acquisition (scanner type and imaging sequence) and pre-processing parameters. We examined how weights from certain layers of our CNN model trained on the source dataset can be used to enhance chronological age classification and regression performance on the target dataset. We also investigated how the contribution of each brain regions' connectivity fingerprints to classification performance changed in different transfer learning conditions.

Data description

We use two datasets in this study; one acquired in our own lab (the in-house dataset) and one that is publicly available (the public dataset). See the Methods section for full details of the data acquisition and pre-processing pipelines.

In-house dataset

A total of 57 subjects with no history of neurological or psychiatric diseases and normal or corrected-to-normal visual acuity participated in the experiment. 28 subjects (14 females) were aged between 20 and 33 years (mean ± SD = 23.9 ± 2.7 years; the young age group) and 29 subjects (14 females) were aged between 59 and 90 years (mean ± SD = 68.7 ± 6.1 years; the elderly age group). Each subject underwent an anatomical scan and a subsequent 600-sec-long resting-state fMRI measurement. Subjects were instructed to lie still while fixating a dark spot in the centre of the screen on a grey background.

Public dataset

We used publicly available data from Consortium for Reliability and Reproducibility (CoRR; [25]): the LMU 1, 2 and 3 datasets [26,27]. Each subject participated in at least two 366-sec-long resting-state fMRI measurements. The datasets contain 209 measurements from 41 subjects (18 females) aged between 20 and 29 years (mean ± SD = 23.8 ± 2.2 years; the young age group) and 146 measurements from 49 subjects (27 females) aged between 55 and 88 years (mean ± SD = 68.6 ± 7.2 years; the elderly age group).

Functional connectivity calculation

To calculate ROI-based whole-brain functional connectivity, we used the Harvard-Oxford Atlas included in FSL [28], consisting of 111 anatomical regions of interest, to obtain 111

meaningful averaged blood-oxygen-level-dependent (BOLD) signals in each measurement. From these 111 time series we calculated full connectivity matrices leading to 111*110/2 = 6105 independent pairwise connectivity features.

Analyses

Classification

We examined whether the classification of age category (young/elderly) based on resting-state functional connectivity data in a relatively small sample (the in-house dataset) can be improved by transferring the knowledge learned on a larger sample (the public dataset). First, we used the in-house dataset for training a connectome-convolutional neural network (CCNN) as well as testing its performance with cross-validation which served as a baseline. Second, we trained the CCNN on the public dataset and used the resulting weights and bias terms either to directly classify the instances in the in-house dataset, or to guide the further training of the network on the in-house dataset. This resulted in 5 different transfer learning conditions (Figure 1.), and the classification performances in these conditions were compared to the baseline, i.e. when the CCNN was trained solely on the in-house dataset. The network architecture and the different training conditions are detailed in the Methods section.

The classification results are summarized in Table 1. Above-chance classification performance was observed (84.2%) when the CCNN was trained exclusively on the in-house dataset ($Conv_{Train}Full_{Train}$). When the CCNN was trained on the public dataset and all the resulting weights were used directly to test the model on the in-house dataset ($Conv_{Const}Full_{Const}$), a slight increase in performance was observed (86%, p = 0.966).Thus, while baseline classification performance is encouraging, there is still room for improvement regarding knowledge transfer.

Importing only the convolutional layers' weights and biases and training the fully connected layers from scratch on the in-house dataset ($Conv_{Const}Full_{Train}$) led to a more pronounced improvement in classification performance (93%), even though the difference to the baseline condition ($Conv_{Train}Full_{Train}$) did not reach the level of significance (p = 0.113). When the weights of the fully connected layers were initialized based on the values learned on the public dataset ($Conv_{Const}Full_{Init}$), a similar result was obtained (91.2%; p = 0.145). Finally, initializing the weights of the convolutional layers based on previously learned values led to a marginally significant improvement in classification performance over the baseline condition (91.2% and p = 0.063 for both $Conv_{Init}Full_{Train}$ and $Conv_{Init}Full_{Init}$). On the whole, training the CCNN on both datasets consistently led to better results than when the model was trained exclusively on one dataset, especially when the convolutional layers were fine-tuned on the target dataset after learning from the source dataset.

Importantly, when the weights learned on the in-house dataset were used to classify instances in the public dataset (Back-transfer), performance dropped dramatically (51.8%). This poor generalization of the representations to the public dataset suggests that the CCNN probably learned specific details of the in-house dataset rather than the general relationship between age and functional connectivity patterns.

ROIs relevant for classification

Based on the learned weights of the first convolutional layer, it is possible to identify which ROIs played an important role in age category classification (see the Methods section and [4]). To this end, we examined the weights learned on the in-house dataset, on the public dataset, or on the in-house dataset by fine-tuning the weights that had been learned previously on the public dataset ($Conv_{Init}Full_{Train}$). From Figure 2. it is evident that weights corresponding to some of the ROIs have relatively high absolute values in most of the filters. This means that

the resting-state functional connectivity fingerprints of these brain regions have an important effect on the output of the first layer of the CCNN.

The top 5 brain regions with the highest absolute value weights are displayed in Figure 3. for each of these three conditions separately. When the CCNN was trained on the in-house dataset, these regions included the posterior division of the left parahippocampal gyrus, the right Heschl's gyrus, caudate, thalamus, and frontal orbital cortex (Figure 3. A). Except for Heschl's gyrus, training the model on the public dataset resulted in different regions, namely the temporooccipital part of the right middle temporal gyrus, the right nucleus accumbens, the temporooccipital part of the left inferior temporal gyrus, and the posterior division of the left temporal fusiform cortex (Figure 3. B). Three of these regions (middle and inferior temporal gyri and the accumbens) continued to have a significant impact when the model was fine-tuned on the in-house dataset after training on the public dataset. The left parahippocampal gyrus, which had the largest impact when training solely on the in-house data, had the second largest weight in this condition. Finally, the temporooccipital part of the right inferior temporal gyrus also played an important role in classification after fine-tuning (Figure 3. C).

Regression

We modified the CCNN model to regress chronological age against functional connectivity patterns (see Methods section for details). When the CCNN was trained solely on the in-house dataset to regress chronological age with the ROIs' functional connectivity fingerprints as independent variables, performance was rather poor (R^2 = 0.5, sMSE = 16.1 years). Using the convolutional layer weights learned on the public dataset for age category classification and then fine-tuning the fully connected layers to perform regression on the in-house data, however, resulted in a remarkable improvement in regression performance (R^2 = 0.66, sMSE = 13.39

years; Figure 4.). The difference in performance between the baseline and transfer learning conditions was significant (p = 0.025).

Discussion

In the present study, we trained a connectome-convolutional neural network to perform binary chronological age category classification (young/elderly) based on region-of-interest-based resting-state functional connectivity patterns derived from fMRI measurements. Even though baseline classification was well above chance, we found that performance could be improved further by training the CCNN model on a larger, publicly available dataset and then making use of the knowledge learned to classify instances in the smaller in-house dataset. This has occurred despite the fact that the two datasets differed considerably in terms of the data acquisition protocol (scanner type and imaging sequence) and pre-processing parameters.

Applying the model trained on the public dataset one-in-one to the in-house dataset resulted in a modest ~2% increase in performance compared to the baseline condition, i.e. when the model was trained exclusively on the in-house data. This suggests that the representations learned on the public dataset are rather abstract and generalize to other datasets reasonably well—nevertheless, classification performance could benefit from continued learning on the target dataset as well. Indeed, allowing the fully connected layers to learn from the in-house dataset—while keeping the weights of the convolutional layers constant—resulted in a marked improvement in performance. Moreover, the most pronounced improvement was observed when the convolutional layers' weights were initialized on the basis of the previously learned values and then trained on the in-house dataset. In general, these results suggest that a handy solution to repurpose existing convolutional neural network models for functional connectivity pattern classification is to fine-tune the convolutional (as well as the fully connected) layers to the target dataset by initializing the weights with the previously learned values. This outcome

bears a close resemblance with previous results in the field of natural image recognition. In particular, [17] found that an eight-layer convolutional neural network trained on a large source image dataset and then fine-tuned to a target dataset shows better generalization that those trained directly on a target dataset of the same size. The authors came to the conclusion that the initialization of network weights with transferred values might be a generally useful method for improving CNN performance, even when the target dataset is large enough to train the network from scratch without overfitting.

Deep learning is a highly promising method for inferring cognitive states and brain pathology from neuroimaging data [2]. In particular, convolutional neural networks have been applied successfully to make predictions on the basis of brain structure [24] and functional connectivity patterns [4]. However, a major drawback of these networks is that their proper training requires extensive amounts of data [6], which substantially exceeds the sample sizes in typical neuroimaging experiments [10]. Performing neuroimaging measurements in the order of thousands to train deep networks from scratch to answer specific research questions under specific data acquisition and processing protocols is impracticable. Nevertheless, with the advent of neuroimaging 'big data' [14], reusing models that have been trained on large-scale datasets seems to be a viable solution to tackle the 'data-hungry' nature of CNNs. This situation is comparable to that in natural image recognition, where large-scale annotated image sets are available (e.g. [9]), and the weights of CNNs trained on such datasets can be transferred effectively to solve visual recognition tasks with limited training data (e.g. [18]). There have been several attempts lately that combined auxiliary datasets for the classification of brain disease states in SVM [29,30] or multinomial regression [31] settings effectively. Recently, [32] used several datasets from different brain imaging studies simultaneously to train a shared multi-layered architecture to decode cognitive states from neural activity patterns. The authors found that aggregating multiple datasets boosted decoding performance on a target dataset, and

this gain in accuracy increased with smaller training size. This offers the potential of learning representations of neural activity from already existing data repositories that can be generalized to newly acquired fMRI data [32]. Our results suggest that transfer learning might be a useful method in applying deep neural networks that have already been trained on larger datasets to fMRI data with a limited number of exemplars. More specifically, the results of the present study also imply that the fine-tuning of convolutional layers by weight initialization is a handy solution to adapt a CNN to the target dataset, at least in the domain of functional connectivity pattern classification.

It is important to note that even though baseline classification performance was well above chance, when the model trained on the in-house dataset was used directly to classify instances in the public dataset, performance dropped to near chance level. This is indicative of a special type of overfitting [12,13]; the CCNN probably learned the idiosyncratic details of the in-house dataset, that correlate with age, but came from our specific measurement parameters instead of the general relationship between functional connectivity patterns and chronological age. This implies that even remarkably good performance should be treated with caution when deep networks are trained on small datasets, and transfer learning might be beneficial under such circumstances as well.

We also investigated which brain regions' connectivity fingerprints played an important role in age category classification in different training conditions, based on the inspection of the learned weights in the first convolutional layer. It turned out that the model relied heavily on two largely different set of regions when training either on the in-house or public datasets. This is consistent with our hyphotheses that convolutional weights learned from the in-house dataset rely on specific measurement circumstances. In the case of the in-house dataset, some of the most important regions, namely the caudate and the thalamus, were located in the proximity of the ventricles. The network might have assigned large weights to these regions due to age-

related ventricular expansion [33] rather than actual alterations in functional connectivity patterns. When the CCNN was first trained on the public dataset and then fine-tuned to the in-house dataset, the importance of these regions decreased. In this case, the most important brain regions turned out to be a combination of the previous two sets, with many of the regions corresponding to those important for classifying instances in the public dataset. It is worth noting that among these regions, the posterior ones, namely the middle and inferior temporal gyri and the parahippocampal gyrus have been shown to reveal alterations in functional connectivity patterns in advanced age [34–36].

Besides age category classification, we also trained our CCNN model to predict chronological age on the basis of brain functional connectivity patterns. When the network was trained exclusively on the in-house dataset, age regression performance was rather poor. Nonetheless, the application of transfer learning lead to a substantial improvement. In particular, keeping the convolutional layer weights that were used successfully in categorization and fine-tuning the fully connected layers to the in-house dataset for the purpose of regression enabled a more accurate prediction of chronological age. Evidence is mounting that brain age—the predicted age of an individual that is derived from neuroimaging data—is related to physical health and brain disease [23]. As such, it is a promising biomarker for individual brain health. Recently, [24] predicted chronological age with less than five years mean absolute error using a CNN trained on T1-weighted structural MRI scans from 2001 healthy individuals. The authors found that even though the within-scanner reliability of brain-predicted age was high, between-scanner reliability was markedly reduced, especially for T1 scans with minimal pre-processing. Thus, multi-center reliability seems to be an issue for CNN-based age estimation, at least when raw structural MRI scans are used for prediction. The precision of age estimation in our study remained well below the one reported by [24]. Regression performance in the transfer learning condition would certainly have benefitted from a larger dataset—the size of the public dataset

was an order of magnitude less than the sample size in [24]. Nevertheless, our results suggest that functional connectivity pattern classification is a promising tool for brain age prediction and deserves further investigation using larger datasets. Moreover, transfer learning, with weight transfer in the convolutional layers and fine-tuning of the fully connected layers to the target dataset, seems to be a plausible solution to adapt CNNs to data acquired with different scanner types and imaging protocols for the purpose of predicting brain age.

Potential implications

We believe that transfer learning has the potential to alleviate the problem of data scarcity regarding deep learning applications in neuroimaging. Here we showed that an already-trained CNN can be fine-tuned effectively to a fMRI functional connectivity dataset with different data acquisition and pre-processing parameters. Since the initial convolutional layers in CNNs tend to learn more general representations [17], it is plausible that models trained on large datasets can also be repurposed to perform a variety of different tasks at relatively low cost.

Methods

In-house data acquisition and pre-processing

Data were acquired on a Siemens Magnetom Prisma 3T MRI scanner (Siemens Healthcare, Erlangen, Germany) at the Brain Imaging Centre, Research Centre for Natural Sciences, Hungarian Academy of Sciences. All head elements of the standard Siemens 64-channel head-neck receiver coil were applied. The protocol consisted of T1-weighted 3D MPRAGE anatomical imaging using 2-fold in-plane GRAPPA acceleration (repetition time (TR)/echo time (TE)/ flip angle (FA) = 2300ms/3ms/9°; field of view (FOV) = 256 mm; isotropic 1 mm spatial resolution). A blipped-CAIPI simultaneous multi-slice (SMS) gradient-echo-EPI sequence [37] was used for functional measurements with 6-fold slice acceleration, using full

brain coverage with an isotropic 2 mm spatial resolution and a TR of 710 ms, without in-plane parallel imaging. A Partial Fourier factor of 7/8 was used to achieve a TE of 30 ms. Image reconstruction was performed using the Slice-GRAPPA algorithm [37] with LeakBlock kernel [38].

Pre-processing of the imaging data was performed using SPM12 [39] and FSL 5.0.9 [40] toolboxes as well as custom-made scripts running on MATLAB 2015a (The MathWorks Inc., Natick, MA, USA). The T2*-weighted functional images were spatially realigned to the first volume for motion correction and coregistered with the T1-weighted anatomical image which was then segmented and normalized to the MNI-152 space using the unified segmentation-normalization tool of SPM12. The resulting grey matter (GM) mask was later used to restrict the analysis of the functional images to GM voxels; while the white matter (WM) and cerebrospinal fluid (CSF) masks were used to extract nuisance signals that are unlikely to reflect neural activity in resting-state time-series. On the realigned and coregistered functional images spatial Independent Component Analysis using FSL's MELODIC ICA 3.14 [41] was performed at the single subject-level to remove artefacts due to an interaction of the multi-slice acquisition with head motion [42].

After the ICA-based cleaning procedure, functional images were normalized to MNI-152 space using deformation field parameters acquired during the segmentation and normalization of the anatomical image, followed by a 5-mm isotropic Gaussian smoothing. After regressing out the head-motion parameters, the mean WM and CSF signals [43], residual time courses from all GM voxels were band-pass filtered using a combination of temporal high-pass (based on the regression of ninth-order discrete cosine transform basis set) and low-pass (bidirectional 12th-order Butterworth IIR) filters to retain signals only within the range of 0.009 and 0.08 Hz [44].

Public data acquisition and pre-processing

The dataset was collected at the Institute of Clinical Radiology, Ludwig-Maximilians-University, Munich, Germany, on a 3 T Philips Achieva scanner (Best, The Netherlands). High-resolution anatomical images were acquired for each subject using a T1-weighted 3D TFE sequence (1 mm isotropic voxels; TR = 2400 ms; FOV = 256 mm; acceleration factor = 2). A total of 120 functional images over 366 sec were collected with a BOLD-sensitive T2* weighted GRE-EPI sequence (4 mm slice thickness with 3 mm x 3 mm in-plane resolution; TR = 3000 ms; TE = 30 ms; FOV = 192 mm). 28 axial slices were acquired in ascending acquisition order covering the whole brain. Further details are available on the website of the datasets [45–47].

Pre-processing of the imaging data was performed using the SPM12 toolbox [39] and custom-made scripts running on MATLAB 2015a (The MathWorks Inc., Natick, MA, USA). Each subject's functional images were motion-corrected, the T2*-weighted functional images in all sessions were spatially realigned to the first volume. Then, the realigned functional images were spatially smoothed using a 5 mm full-width half maximum Gaussian filter. The T1-weighted anatomical images in each session were coregistered to the mean T2*-weighted functional images created during the realignment step. The coregistered anatomical images were segmented using the unified segmentation and normalization tool of SPM12. The resulting GM mask was later used to restrict the analysis of the functional images to GM voxels; while the WM and CSF masks were used to extract nuisance signals that are unlikely to reflect neural activity in resting-state time-series. The realigned functional images were normalized to the MNI-152 space using deformation field parameters generated during the segmentation and normalization of the anatomical images. After regressing out the head-motion parameters, the mean WM, CSF, and whole-brain signals [43], residual time courses from all GM voxels were band-pass filtered using a combination of temporal high-pass (based on the regression of ninth-

order discrete cosine transform basis set) and low-pass (bidirectional 12th-order Butterworth IIR) filters to retain signals only within the range of 0.009 and 0.08 Hz [44].

Connectome-convolutional neural network architecture

We used a slightly modified version of our connectome-convolutional neural network model that has previously proved successful in the classification of functional connectivity patterns [4]. In detail, we arranged the connectivity features into 111 × 111 matrices (corresponding to the 111 ROIs) and applied line-by-line convolution (filter size: 1 × 111) followed by convolution by column (filter size: 111 × 1). Thus, we treated the connectivity fingerprint of each ROI (rows in the input matrix) as a unit whose weights can be shared across the whole connectivity matrix. This is based on the assumption that the learned convolutional filter will assign large weights to ROIs that show altered connectivity between the age groups, and thus connectivity strength with those altered regions will have a large influence on the output [4].

In the first convolutional layer we trained 64 filters, i.e. 64 differently weighted sums of each ROI's connectivity fingerprint were calculated. In the second convolutional layer we trained 256 filters. The output of this layer is fed into a fully connected hidden layer with 96 neurons that are connected to the output layer consisting of two neurons corresponding to the two classes. We applied rectified linear unit (ReLU; [48]) non-linearity in the convolutional neural network and the softmax function [49] on the output layer to calculate the probability of each instance belonging to a certain class. The network is trained with cross-entropy as a loss function [6]. To train a robust classifier, we applied dropout regularization [50,51] with keeping probability of 0.6 and an Adam optimizer [52] with a learning rate of 0.001 and 5000 training iterations. The two convolutional layers of the CCNN model include 111*64+111*64*256 = 1825728 trainable weights and 320 bias terms. The fully connected hidden and output layers include 256*96+96*2 = 24768 trainable weights and 98 bias terms.

The CCNN model was implemented in Python using TensorFlow.

Transfer learning for classification

To establish the baseline classification performance (i.e. when the in-house dataset is used for both training and testing), we applied cross-validation. Measurements from the 57 subjects were randomly divided into 10 folds. Measurements in each fold constituted the test set for that fold with five or six subjects while the remaining measurements constituted the training set. This same partitioning was used to evaluate all classifiers and conditions. As in this case, the convolutional as well as the fully connected layers were trained in each fold, we refer to this condition as $Conv_{Train}Full_{Train}$.

To evaluate the transfer of weights and bias terms from the public to the in-house dataset, the CCNN was trained on all instances of the public dataset in one fold. Measurements in the in-house dataset were omitted from training. Subsequently, weights and bias terms learned on the public dataset were transferred to classify instances in the in-house dataset. In one condition, the resulting weights and bias terms of the convolutional as well as the fully connected layers were used to classify each instance in the in-house dataset. Since all layers' weights and biases terms were constants based on what had been learned on the public dataset previously, we refer to this condition as $Conv_{Const}Full_{Const}$. In another condition, after training the CCNN on all instances of the public dataset, the model was further trained and evaluated on the in-house dataset using the 10-fold cross-validation scheme. At this stage, the weights and bias terms of the two convolutional layers were kept constant while those of the fully connected layers were newly initialized (using 'Xavier' initialization; [53]) and trained in each fold of the cross-validation ($Conv_{Const}Full_{Train}$).

To examine whether the representations learned on the smaller in-house dataset can be generalized to the larger dataset, we transferred weights and biases learned on the in-house

dataset to classify instances in the public dataset. We refer to this condition as *Back-transfer*, as in this case, the direction of knowledge transfer is the opposite to that in the rest of the conditions. In particular, this condition is the exact opposite of the $Conv_{Const}Full_{Const}$ condition, inasmuch as the weights of the convolutional and fully connected layers learned on the in-house dataset are used as constants when testing on the public dataset. Performance in this condition is supposed indicate whether the representations learned on such a small dataset are general in the sense that they concern the relationship between functional connectivity and age, or specific to the characteristic features of the in-house dataset.

We also examined the effect of weight initialization based on the public dataset. Similarly to the previous ones, the conditions described hereinafter involve the training of the CCNN on all instances of the public dataset as a first step. In the second step, instead of keeping the weights and bias terms learned on the public dataset constant, their values are used to re-initialize the same weights and bias terms for training on the in-house dataset in each fold of the cross-validation. In one condition, all layers' weights and bias terms were initialized based on what had been learned previously on the public dataset ($Conv_{Init}Full_{Init}$). In a further condition, only the convolutional layers' parameters were initialized based on the previously learned values, while the weights and biases of the fully connected layers were newly initialized in each fold using 'Xavier' initialization ($Conv_{Init}Full_{Train}$). Finally, we examined performance in a condition in which the weights and biases of the convolutional layers were kept constant while those of the fully connected layers were initialized using the values learned on the public dataset ($Conv_{Const}Full_{Init}$).

We assessed classification performance with two metrics: accuracy (the proportion of correctly classified instances) and area under the receiver operating characteristic curve (AUC). Classification performance in each transfer learning condition was compared separately to the

performance obtained in the baseline condition using a binomial test [54]. We consider the difference significant if the calculated p-value is lower than 0.05.

Evaluating the role of ROIs in classification

To examine the impact of each ROI's connectivity fingerprint on classification outcome, the absolute values of the final weights of the 64 filters in the first convolutional layer were summed for each ROI separately. High values represent ROIs that have large weights in most filters and hence their resting-state functional connectivity patterns play an important role in determining the output of the first layer. ROIs with the highest values are displayed on representative sections of the MNI brain template for weights learned on the public and in-house datasets.

Transfer learning for regression

The CCNN was modified to implement a regression model with the functional connectivity fingerprints of ROIs as independent variables and chronological age as the dependent variable. To this end, the number of neurons in the output layer was reduced to one. The total number of trainable weights in the fully connected layers changed accordingly to 24672 plus 97 bias terms. To train the network, we used mean squared error as the loss function and Adam optimizer with a learning rate of 0.0005 and 15000 training iterations. Dropout regularization with a keeping probability of 0.6 was applied. Baseline regression performance was established using the in-house dataset and a 10-fold cross-validation scheme.

To examine how transfer learning from the public dataset aids regression when using the in-house data, the convolutional layer weights and bias terms that had been learned previously on the public dataset to perform binary age category classification were used as constants. First, the fully connected layers of the CCNN were trained on the public dataset to regress chronological age. Second, the fully connected layers were trained to perform regression on

the in-house dataset. In this second step, the weights and biases of the fully connected layers were initialized with the values learned on the public dataset in the previous step. Regression performance on the in-house dataset was established using the 10-fold cross-validation scheme.

We evaluated regression performance using the coefficient of determination (R^2) and the square root of the mean squared error (sMSE). A paired t-test on predicted chronological ages was performed to compare regression performances in the baseline and transfer learning conditions.

Availability of supporting data and materials

The T1-weighted and T2*-weighted MRI scans, connectivity matrices, and labels are available in the GigaScience repository GigaDB.

Availability of source code and requirements

Project name: Transfer learning for CCNN-based resting-state functional connectivity pattern analysis

Project home page: https://github.com/vaklip/transfer_learning_ccnn

Operating system(s): Platform independent

Programming language: Python

Other requirements: TensorFlow

License: MIT

Declarations

List of abbreviations

AUC: area under the receiver operating characteristic curve; BOLD: blood-oxygen-level-dependent; CCNN: connectome-convolutional neural network; CNN: convolutional neural network; CSF: cerebrospinal fluid; fMRI: functional magnetic resonance imaging; GM: grey matter; ReLU: rectified linear unit; ROI: region of interest; sMSE: square root of the mean squared error; SVM: support vector machine; WM: white matter

Ethics, consent and permissions

Participants gave informed written consent in accordance with the protocols approved by Health Registration and Training Center (ENKK 006641/2016/OTIG), Budapest, Hungary.

Competing interests

The authors declare that they have no competing interests.

Funding

This work was supported by a grant from the Hungarian Brain Research Program (KTIA_13_NAP–A–I/18) to ZV.

Authors’ contributions

P.V., R.J.D.M, and Z.V. designed the experiment; P.V., R.J.D.M, and P.H. collected the imaging data and performed the data pre-processing; P.V. and R.J.D.M developed the convolutional neural network and the transfer learning protocols and performed the statistical analysis; all authors contributed to the interpretation of the results; P.V. and R.J.D.M. wrote the manuscript with the assistance of Z.V. and P.H.

Acknowledgements

We thank Petra Madurka and Annamária Manga for their assistance with data collection. We are very grateful to Tibor Auer for his assistance in data pre-processing.

References

1. LeCun Y, Bengio Y, Hinton G. Deep learning. Nature. 2015;521:nature14539.

2. Cohen JD, Daw N, Engelhardt B, Hasson U, Li K, Niv Y, et al. Computational approaches to fMRI analysis. Nat Neurosci. 2017;20:nn.4499.

3. Vieira S, Pinaya WHL, Mechelli A. Using deep learning to investigate the neuroimaging correlates of psychiatric and neurological disorders: Methods and applications. Neurosci Biobehav Rev. 2017;74:58–75.

4. Meszlényi RJ, Buza K, Vidnyánszky Z. Resting State fMRI Functional Connectivity-Based Classification Using a Convolutional Neural Network Architecture. Front Neuroinformatics [Internet]. 2017 [cited 2017 Nov 13];11. Available from: https://www.frontiersin.org/articles/10.3389/fninf.2017.00061/full

5. Marblestone AH, Wayne G, Kording KP. Toward an Integration of Deep Learning and Neuroscience. Front Comput Neurosci [Internet]. 2016 [cited 2017 Nov 20];10. Available from: https://www.ncbi.nlm.nih.gov/pmc/articles/PMC5021692/

6. Goodfellow I, Bengio Y, Courville A. Deep learning. MIT press; 2016.

7. Krizhevsky A, Sutskever I, Hinton GE. ImageNet Classification with Deep Convolutional Neural Networks. In: Pereira F, Burges CJC, Bottou L, Weinberger KQ, editors. Adv Neural Inf Process Syst 25 [Internet]. Curran Associates, Inc.; 2012 [cited 2017 Nov 21]. p. 1097–1105. Available from: http://papers.nips.cc/paper/4824-imagenet-classification-with-deep-convolutional-neural-networks.pdf

8. Russakovsky O, Deng J, Su H, Krause J, Satheesh S, Ma S, et al. ImageNet Large Scale Visual Recognition Challenge. Int J Comput Vis. 2015;115:211–52.

9. The ImageNet database. [Internet]. [cited 2018 Jan 22]. Available from: http://image-net.org/

10. Arbabshirani MR, Plis S, Sui J, Calhoun VD. Single subject prediction of brain disorders in neuroimaging: Promises and pitfalls. NeuroImage. 2017;145:137–65.

11. Xia M, He Y. Functional connectomics from a “big data” perspective. NeuroImage. 2017;160:152–67.

12. Lemm S, Blankertz B, Dickhaus T, Müller K-R. Introduction to machine learning for brain imaging. NeuroImage. 2011;56:387–99.

13. Pereira F, Mitchell T, Botvinick M. Machine learning classifiers and fMRI: a tutorial overview. NeuroImage. 2009;45:S199–209.

14. Poldrack RA, Gorgolewski KJ. Making big data open: data sharing in neuroimaging. Nat Neurosci. 2014;17:1510–7.

15. Glover GH, Mueller BA, Turner JA, van Erp TGM, Liu TT, Greve DN, et al. Function biomedical informatics research network recommendations for prospective multicenter functional MRI studies. J Magn Reson Imaging. 2012;36:39–54.

16. Pan SJ, Yang Q. A Survey on Transfer Learning. IEEE Trans Knowl Data Eng. 2010;22:1345–59.

17. Yosinski J, Clune J, Bengio Y, Lipson H. How transferable are features in deep neural networks? In: Ghahramani Z, Welling M, Cortes C, Lawrence ND, Weinberger KQ, editors. Adv Neural Inf Process Syst 27 [Internet]. Curran Associates, Inc.; 2014 [cited 2017 Nov 28]. p. 3320–3328. Available from: http://papers.nips.cc/paper/5347-how-transferable-are-features-in-deep-neural-networks.pdf

18. Oquab M, Bottou L, Laptev I, Sivic J. Learning and Transferring Mid-Level Image Representations using Convolutional Neural Networks. 2014 [cited 2017 Nov 28]. p. 1717–24. Available from: https://www.cv-foundation.org/openaccess/content_cvpr_2014/html/Oquab_Learning_and_Transferring_2014_CVPR_paper.html

19. Donahue J, Jia Y, Vinyals O, Hoffman J, Zhang N, Tzeng E, et al. DeCAF: A Deep Convolutional Activation Feature for Generic Visual Recognition. PMLR [Internet]. 2014 [cited 2017 Nov 28]. p. 647–55. Available from: http://proceedings.mlr.press/v32/donahue14.html

20. Sharif Razavian A, Azizpour H, Sullivan J, Carlsson S. CNN Features Off-the-Shelf: An Astounding Baseline for Recognition. 2014 [cited 2017 Nov 28]. p. 806–13. Available from: https://www.cv-foundation.org/openaccess/content_cvpr_workshops_2014/W15/html/Razavian_CNN_Features_Off-the-Shelf_2014_CVPR_paper.html

21. Zeiler MD, Fergus R. Visualizing and Understanding Convolutional Networks. In: Fleet D, Pajdla T, Schiele B, Tuytelaars T, editors. Comput Vis – ECCV 2014 [Internet]. Cham: Springer International Publishing; 2014 [cited 2017 Nov 28]. p. 818–33. Available from: http://link.springer.com/10.1007/978-3-319-10590-1_53

22. Yosinski J, Clune J, Nguyen A, Fuchs T, Lipson H. Understanding Neural Networks Through Deep Visualization. ArXiv150606579 Cs [Internet]. 2015 [cited 2017 Nov 29]; Available from: http://arxiv.org/abs/1506.06579

23. Cole JH, Franke K. Predicting Age Using Neuroimaging: Innovative Brain Ageing Biomarkers. Trends Neurosci. 2017;40:681–90.

24. Cole JH, Poudel RPK, Tsagkrasoulis D, Caan MWA, Steves C, Spector TD, et al. Predicting brain age with deep learning from raw imaging data results in a reliable and heritable biomarker. NeuroImage. 2017;163:115–24.

25. Zuo X-N, Anderson JS, Bellec P, Birn RM, Biswal BB, Blautzik J, et al. An open science resource for establishing reliability and reproducibility in functional connectomics. Sci Data. 2014;1:sdata201449.

26. Blautzik J, Keeser D, Berman A, Paolini M, Kirsch V, Mueller S, et al. Long-Term Test-Retest Reliability of Resting-State Networks in Healthy Elderly Subjects and Patients with Amnestic Mild Cognitive Impairment. J Alzheimers Dis. 2013;34:741–54.

27. Blautzik J, Vetter C, Peres I, Gutyrchik E, Keeser D, Berman A, et al. Classifying fMRI-derived resting-state connectivity patterns according to their daily rhythmicity. NeuroImage. 2013;71:298–306.

28. Templates and Atlases included with FSL. [Internet]. [cited 2018 Jan 22]. Available from: https://fsl.fmrib.ox.ac.uk/fsl/fslwiki/Atlases

29. Cheng B, Liu M, Suk H-I, Shen D, Zhang D, Initiative ADN. Multimodal manifold-regularized transfer learning for MCI conversion prediction. Brain Imaging Behav. 2015;9:913–26.

30. Cheng B, Liu M, Shen D, Li Z, Zhang D, Initiative the ADN. Multi-Domain Transfer Learning for Early Diagnosis of Alzheimer’s Disease. Neuroinformatics. 2017;15:115–32.

31. Wachinger C, Reuter M. Domain adaptation for Alzheimer’s disease diagnostics. NeuroImage. 2016;139:470–9.

32. Mensch A, Mairal J, Bzdok D, Thirion B, Varoquaux G. Learning Neural Representations of Human Cognition across Many fMRI Studies. In: Guyon I, Luxburg UV, Bengio S, Wallach H, Fergus R, Vishwanathan S, et al., editors. Adv Neural Inf Process Syst 30 [Internet]. Curran Associates, Inc.; 2017 [cited 2017 Dec 12]. p. 5885–5895. Available from: http://papers.nips.cc/paper/7170-learning-neural-representations-of-human-cognition-across-many-fmri-studies.pdf

33. Fjell AM, Walhovd KB. Structural Brain Changes in Aging: Courses, Causes and Cognitive Consequences. Rev Neurosci. 2011;21:187–222.

34. Achard S, Bullmore E. Efficiency and Cost of Economical Brain Functional Networks. PLoS Comput Biol. 2007;3:e17.

35. Damoiseaux JS, Beckmann CF, Arigita EJS, Barkhof F, Scheltens P, Stam CJ, et al. Reduced resting-state brain activity in the “default network” in normal aging. Cereb Cortex. 2008;18:1856–64.

36. Onoda K, Ishihara M, Yamaguchi S. Decreased Functional Connectivity by Aging Is Associated with Cognitive Decline. J Cogn Neurosci. 2012;24:2186–98.

37. Setsompop K, Gagoski BA, Polimeni JR, Witzel T, Wedeen VJ, Wald LL. Blipped-controlled aliasing in parallel imaging for simultaneous multislice echo planar imaging with reduced g-factor penalty. Magn Reson Med. 2012;67:1210–24.

38. Cauley SF, Polimeni JR, Bhat H, Wald LL, Setsompop K. Interslice leakage artifact reduction technique for simultaneous multislice acquisitions. Magn Reson Med. 2014;72:93–102.

39. SPM12 By members & collaborators of the Wellcome Trust Centre for Neuroimaging. [Internet]. [cited 2018 Jan 26]. Available from: http://www.fil.ion.ucl.ac.uk/spm/software/spm12/

40. FMRIB Software Library v5.0 Created by the Analysis Group, FMRIB, Oxford, UK. [Internet]. [cited 2018 Jan 26]. Available from: https://fsl.fmrib.ox.ac.uk/fsl/fslwiki

41. Beckmann CF, Smith SM. Probabilistic independent component analysis for functional magnetic resonance imaging. IEEE Trans Med Imaging. 2004;23:137–52.

42. Griffanti L, Douaud G, Bijsterbosch J, Evangelisti S, Alfaro-Almagro F, Glasser MF, et al. Hand classification of fMRI ICA noise components. NeuroImage. 2017;154:188–205.

43. Weissenbacher A, Kasess C, Gerstl F, Lanzenberger R, Moser E, Windischberger C. Correlations and anticorrelations in resting-state functional connectivity MRI: A quantitative comparison of preprocessing strategies. NeuroImage. 2009;47:1408–16.

44. Cordes D, Haughton VM, Arfanakis K, Carew JD, Turski PA, Moritz CH, et al. Frequencies Contributing to Functional Connectivity in the Cerebral Cortex in “Resting-state” Data. Am J Neuroradiol. 2001;22:1326–33.

45. LMU 1 dataset. [Internet]. [cited 2018 Jan 26]. Available from: http://fcon_1000.projects.nitrc.org/indi/CoRR/html/lmu_1.html

46. LMU 2 dataset. [Internet]. [cited 2018 Jan 26]. Available from: http://fcon_1000.projects.nitrc.org/indi/CoRR/html/lmu_2.html

47. LMU 3 dataset. [Internet]. [cited 2018 Jan 26]. Available from: http://fcon_1000.projects.nitrc.org/indi/CoRR/html/lmu_3.html

48. Nair V, Hinton GE. Rectified Linear Units Improve Restricted Boltzmann Machines. Proc 27th Int Conf Int Conf Mach Learn [Internet]. USA: Omnipress; 2010. p. 807–814. Available from: http://dl.acm.org/citation.cfm?id=3104322.3104425

49. Bridle JS. Probabilistic Interpretation of Feedforward Classification Network Outputs, with Relationships to Statistical Pattern Recognition. Neurocomputing [Internet]. Springer, Berlin, Heidelberg; 1990 [cited 2017 Nov 13]. p. 227–36. Available from: https://link.springer.com/chapter/10.1007/978-3-642-76153-9_28

50. Srivastava N, Hinton G, Krizhevsky A, Sutskever I, Salakhutdinov R. Dropout: A Simple Way to Prevent Neural Networks from Overfitting. J Mach Learn Res. 2014;15:1929–1958.

51. Wager S, Wang S, Liang PS. Dropout Training as Adaptive Regularization. In: Burges CJC, Bottou L, Welling M, Ghahramani Z, Weinberger KQ, editors. Adv Neural Inf Process Syst 26 [Internet]. Curran Associates, Inc.; 2013. p. 351–359. Available from: http://papers.nips.cc/paper/4882-dropout-training-as-adaptive-regularization.pdf

52. Kingma DP, Ba J. Adam: A Method for Stochastic Optimization. ArXiv14126980 Cs [Internet]. 2014 [cited 2018 Jan 22]; Available from: http://arxiv.org/abs/1412.6980

53. Glorot X, Bengio Y. Understanding the difficulty of training deep feedforward neural networks. PMLR [Internet]. 2010 [cited 2017 Nov 13]. p. 249–56. Available from: http://proceedings.mlr.press/v9/glorot10a.html

54. Salzberg SL. On Comparing Classifiers: Pitfalls to Avoid and a Recommended Approach. Data Min Knowl Discov. 1997;1:317–28.

Figure 1. Schematic illustration of the baseline, transfer learning, and back-transfer conditions. Rectangles represent the weights and bias terms in each layer. The color of the rectangles specifies which dataset the layer was originally trained on (green: in-house dataset, blue: public dataset). Open and closed padlocks indicate whether the weights and bias terms were used for initialization or kept constant, respectively. The color of the input and output signs indicate which dataset was used for testing (the target dataset). Subscripts in the condition names indicate whether the weights and biases in the respective layers were kept constant (Const), initialized on previously learned values (Init), or learned from scratch (Train) when the CCNN was applied to the target dataset. See the Methods section for details.

Table 1. Performance measures of the baseline and transfer learning conditions.

Classification	$Conv_{Train}$ $Full_{Train}$	$Conv_{Const}$ $Full_{Const}$	$Conv_{Const}$ $Full_{Train}$	$Conv_{Init}$ $Full_{Init}$	$Conv_{Init}$ $Full_{Train}$	$Conv_{Const}$ $Full_{Init}$	Back-transfer
Accuracy (%)	84.2	86.0	93.0	91.2	91.2	91.2	51.8
AUC	0.936	0.966	0.962	0.946	0.946	0.952	0.669

Figure 2. Learned weights of the first convolutional layer of the CCNN model trained for age category classification. This layer has 64*111 weights. To determine which ROIs play an important role in classification, we summed the absolute value of the weights across the 64 filters for each ROI separately. High values represent ROIs that have large absolute weights in most filters. A) Weights learned on the in-house dataset. B) Weights learned on the public

dataset. C) Weights learned on the in-house dataset by fine-tuning the weights that had been learned previously on the public dataset ($Conv_{Init}Full_{Train}$).

Figure 3. Most influential brain regions in age category classification based on the weights of the first convolutional layer of the CCNN. A) Weights are learned exclusively on the in-house dataset. B) Weights are learned exclusively on the public dataset. C) Weights are learned on the in-house dataset after initializing with the values learned previously on the public dataset ($Conv_{Init}Full_{Train}$).

Figure 4. Chronological age regression performance with (blue) and without (green) transfer learning. Histograms show the distribution of errors in predicted chronological age in years.

Figure 1

Click here to download Figure Figure1.pdf

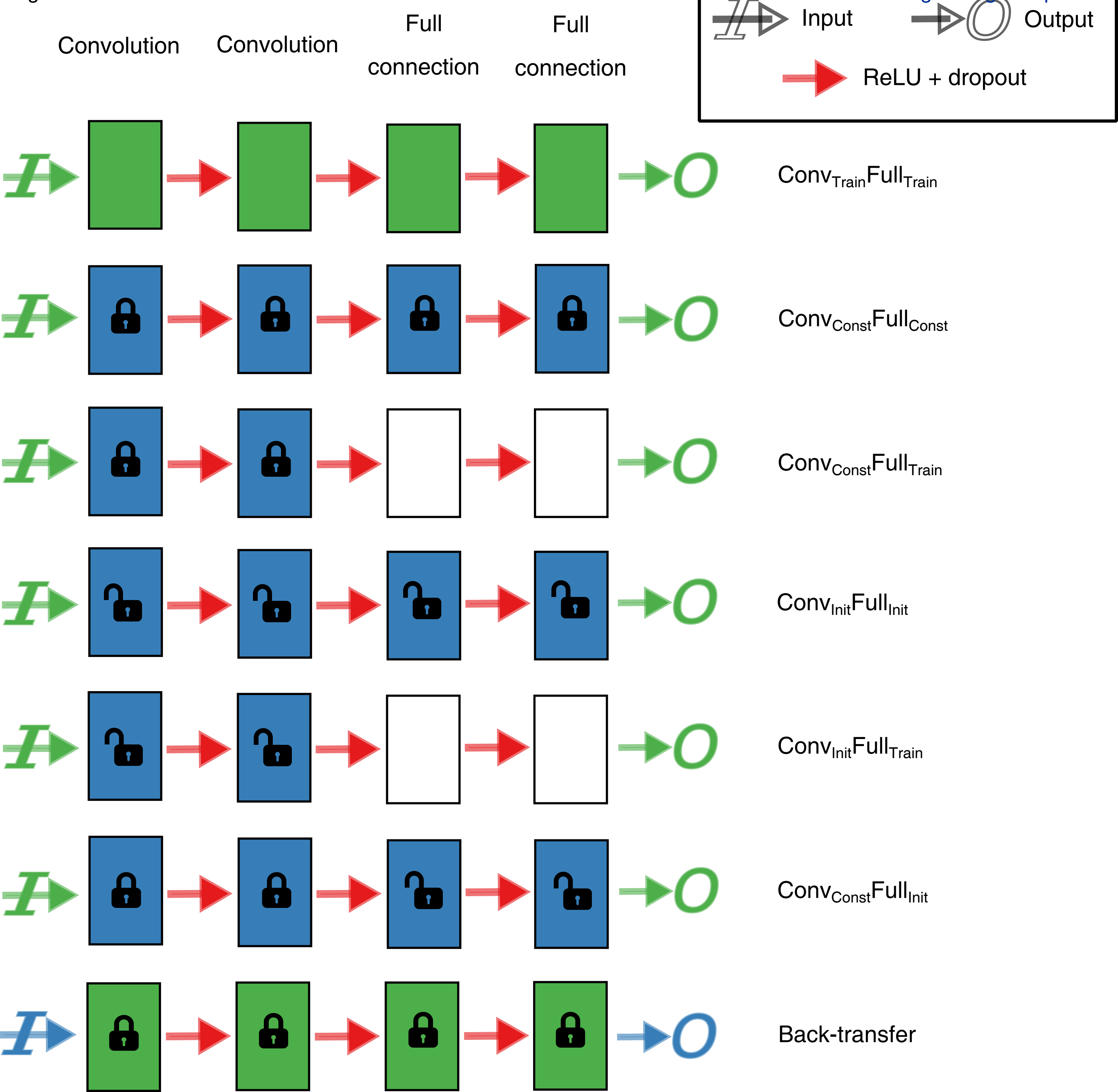

Figure 2

Click here to download Figure Figure2.pdf

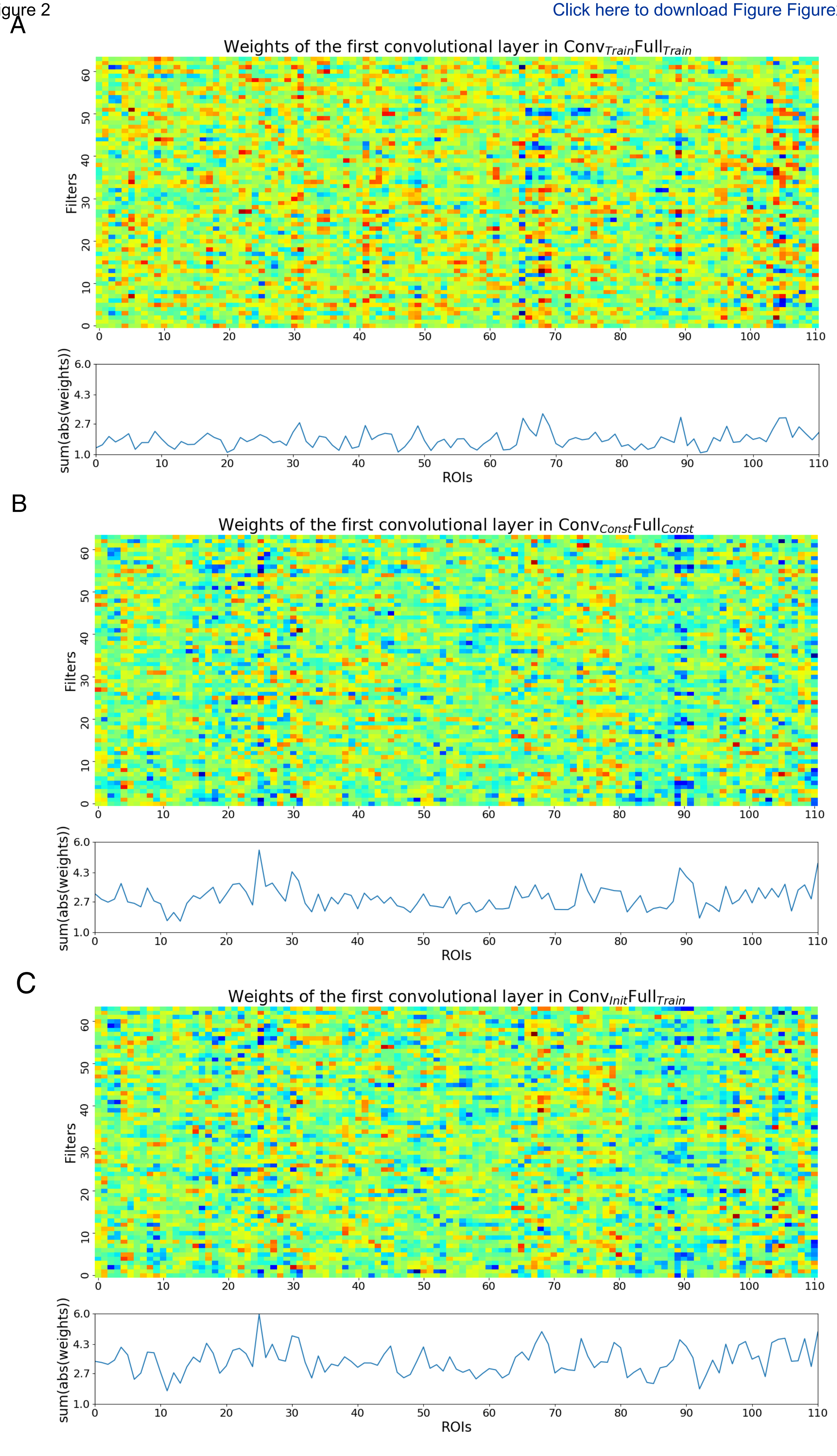

Figure 3 Click here to download Figure Figure3.pdf

A

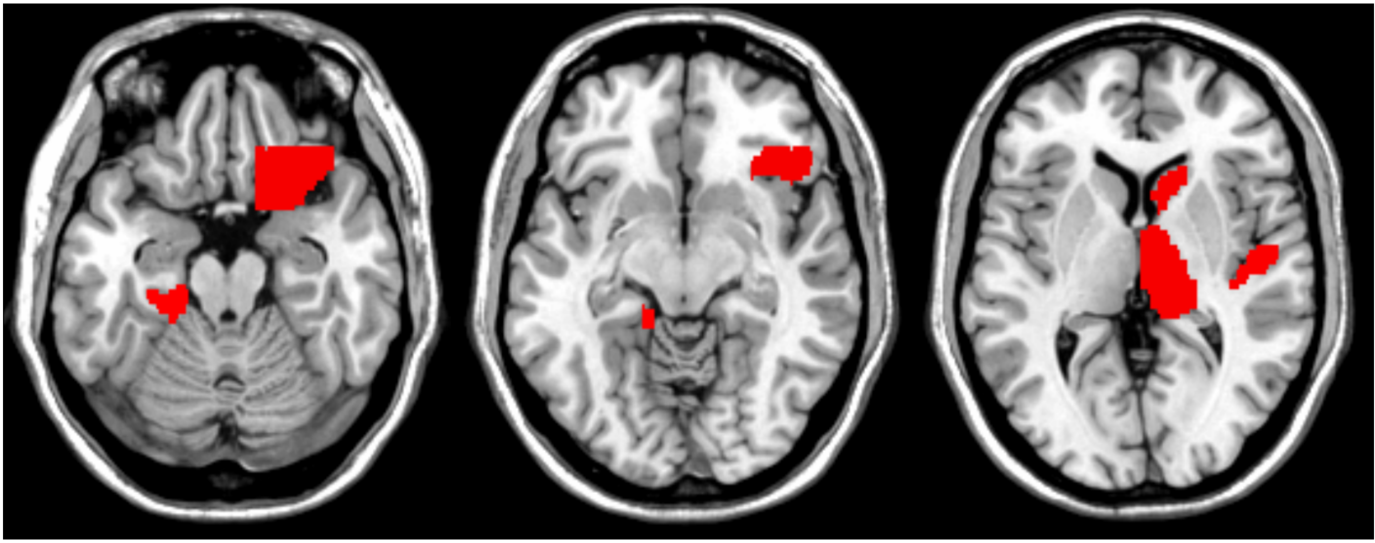

B

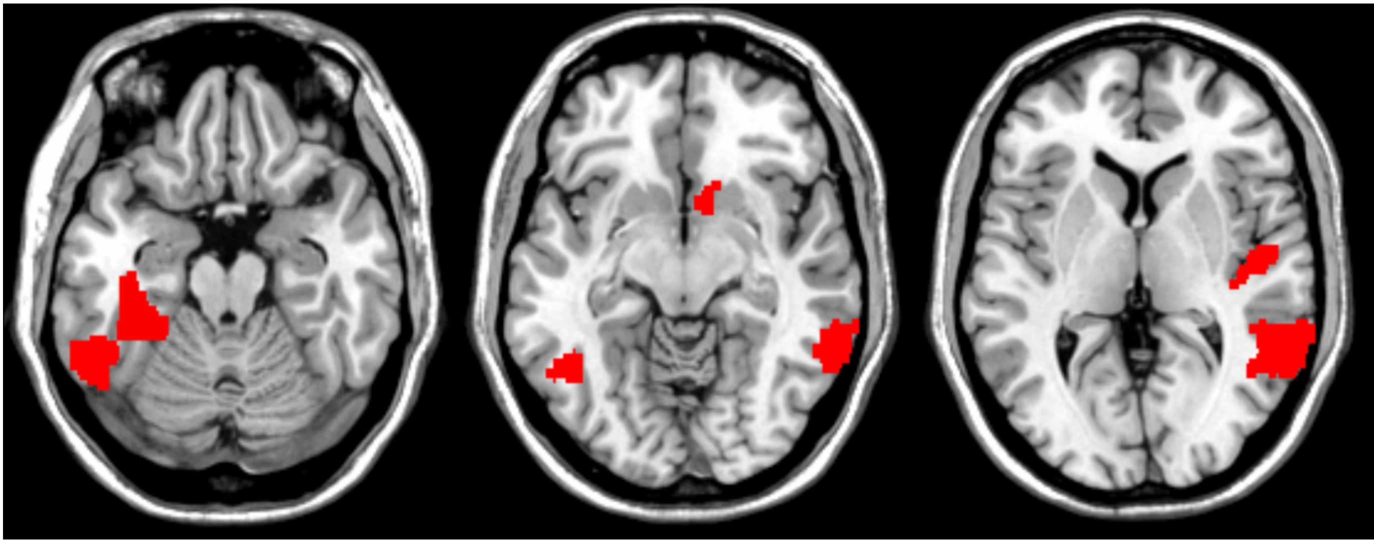

C

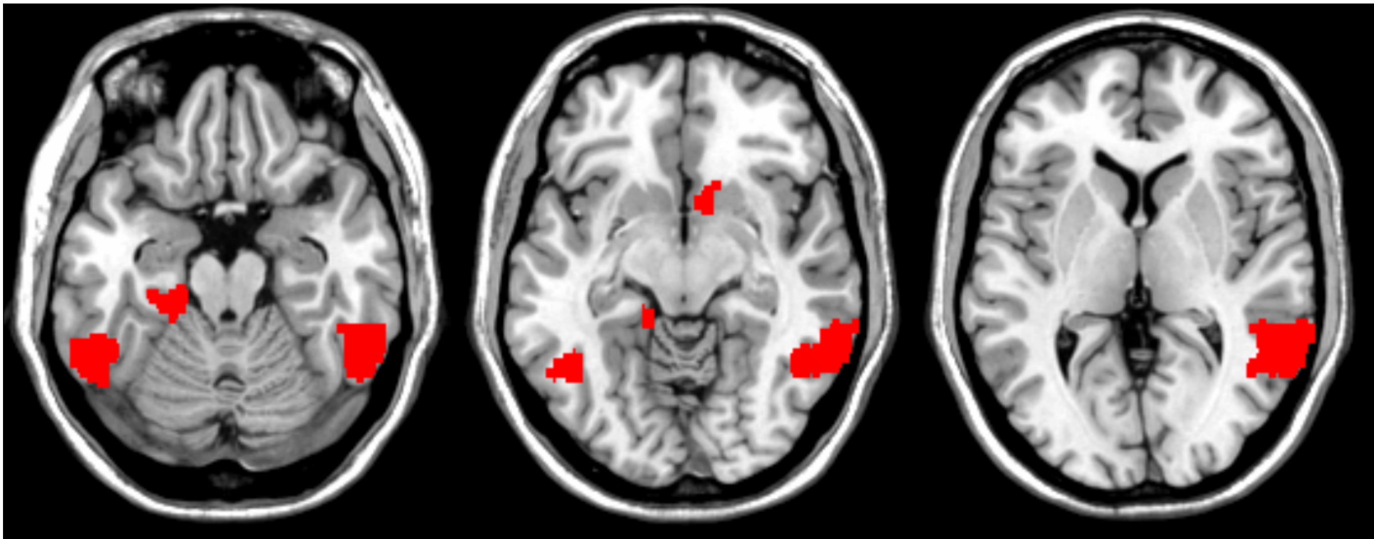

Figure 4

Click here to download Figure Figure4.pdf

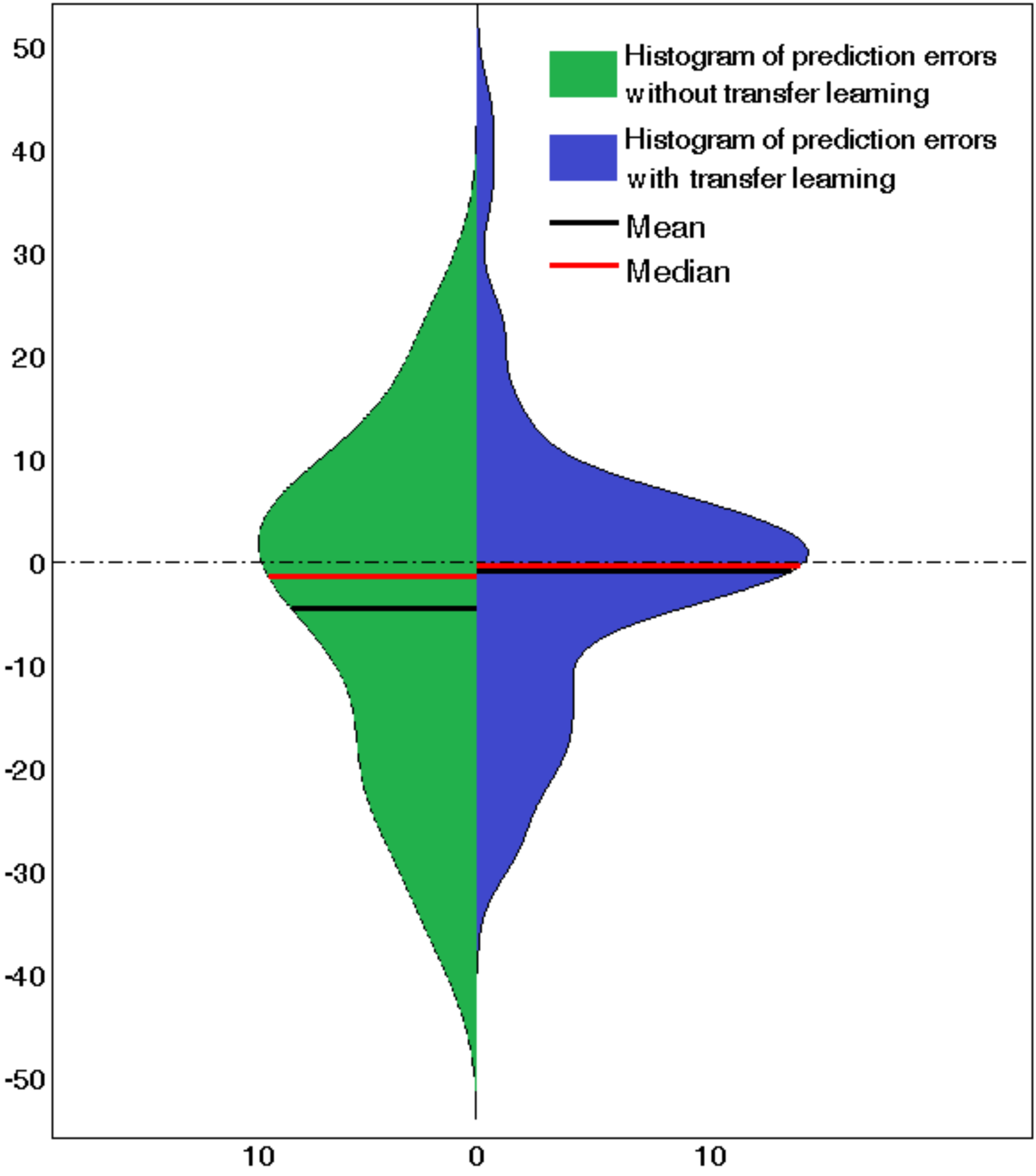

Supplement: GIGA-D-18-00100_Original_Submission.pdf [file giy130_giga-d-18-00100_original_submission.pdf]
