# Supplementary material for: Transfer learning improves resting-state functional connectivity pattern analysis using convolutional neural networks
Source: Gigascience. 2018 Nov 5;7(12):giy130. doi: 10.1093/gigascience/giy130 (PMC6283213; doi:10.1093/gigascience/giy130)
Supplement: GIGA-D-18-00100_Revision_2.pdf [file giy130_giga-d-18-00100_revision_2.pdf]

GigaScience

Transfer learning improves resting-state functional connectivity pattern analysis using convolutional neural networks

--Manuscript Draft--

**Manuscript Number:**	GIGA-D-18-00100R2
**Full Title:**	Transfer learning improves resting-state functional connectivity pattern analysis using convolutional neural networks
**Article Type:**	Research
**Funding Information:**	Hungarian Brain Research Program (KTIA_13_NAP–A–I/18) — Dr. Zoltán Vidnyánszky
**Abstract:**	Background<br>Deep learning is gaining importance in the prediction of cognitive states and brain pathology based on neuroimaging data. Including multiple hidden layers in artificial neural networks enables unprecedented predictive power; however, the proper training of deep neural networks requires thousands of exemplars. Collecting this amount of data is not feasible in typical neuroimaging experiments. A handy solution to this problem, which has largely fallen outside the scope of deep learning applications in neuroimaging, is to repurpose deep networks that have already been trained on large datasets by fine-tuning them to target datasets/tasks with fewer exemplars. Here we investigated how this method, called transfer learning, can aid age category classification and regression based on brain functional connectivity patterns derived from resting-state functional magnetic resonance imaging. We trained a connectome-convolutional neural network on a larger public dataset and then examined how the knowledge learned can be used effectively to perform these tasks on smaller target datasets collected with a different type of scanner and/or imaging protocol and pre-processing pipeline.<br>Results<br>Age classification on the target datasets benefitted from transfer learning. Significant improvement (~9-13% increase in accuracy) was observed when the convolutional layers' weights were initialized based on the values learned on the public dataset and then fine-tuned to the target datasets. Transfer learning also appeared promising in improving the otherwise poor prediction of chronological age.<br>Conclusions<br>Transfer learning is a plausible solution to adapt convolutional neural networks to neuroimaging data with few exemplars and different data acquisition and pre-processing protocols.
**Corresponding Author:**	Pál Vakli, Ph.D.<br><br>HUNGARY
**Corresponding Author Secondary Information:**	
**Corresponding Author's Institution:**	
**Corresponding Author's Secondary Institution:**	
**First Author:**	Pál Vakli, Ph.D.
**First Author Secondary Information:**	
**Order of Authors:**	Pál Vakli, Ph.D.<br>Regina Júlia Deák-Meszlényi<br>Petra Hermann, Ph.D.<br>Zoltán Vidnyánszky, D.Sc.
**Order of Authors Secondary Information:**	
**Response to Reviewers:**	Please see our responses to the comments attached in a separate file.

Powered by Editorial Manager® and ProduXion Manager® from Aries Systems Corporation

Additional Information:	
Question	Response
Are you submitting this manuscript to a special series or article collection?	No
**Experimental design and statistics**<br><br>Full details of the experimental design and statistical methods used should be given in the Methods section, as detailed in our Minimum Standards Reporting Checklist. Information essential to interpreting the data presented should be made available in the figure legends.<br><br>Have you included all the information requested in your manuscript?	Yes
**Resources**<br><br>A description of all resources used, including antibodies, cell lines, animals and software tools, with enough information to allow them to be uniquely identified, should be included in the Methods section. Authors are strongly encouraged to cite Research Resource Identifiers (RRIDs) for antibodies, model organisms and tools, where possible.<br><br>Have you included the information requested as detailed in our Minimum Standards Reporting Checklist?	Yes
**Availability of data and materials**<br><br>All datasets and code on which the conclusions of the paper rely must be either included in your submission or deposited in publicly available repositories (where available and ethically appropriate), referencing such data using a unique identifier in the references and in the "Availability of Data and Materials" section of your manuscript.	Yes

Have you have met the above requirement as detailed in our Minimum Standards Reporting Checklist?	

Powered by Editorial Manager® and ProduXion Manager® from Aries Systems Corporation

Manuscript

Click here to view linked References

Click here to access/download;Manuscript;Transfer_learning_manuscript.pd

Transfer learning improves resting-state functional connectivity pattern analysis using convolutional neural networks

Pál Vakli[1*†], Regina J. Deák-Meszlényi[1,2*], Petra Hermann[1], Zoltán Vidnyánszky[1,2]

[1]Brain Imaging Centre, Research Centre for Natural Sciences, Hungarian Academy of Sciences, Magyar tudósok körútja 2., 1117 Budapest, Hungary

[2]Department of Cognitive Science, Budapest University of Technology and Economics, Egry József utca 1., 1111 Budapest, Hungary

*Pál Vakli and Regina J. Deák-Meszlényi contributed equally to this paper.

†Correspondence: Pál Vakli, Brain Imaging Centre, Research Centre for Natural Sciences, Hungarian Academy of Sciences, Magyar tudósok körútja 2., 1117 Budapest, Hungary. Tel: +361 3826446; E-mail: vakli.pal@ttk.mta.hu

E-mail addresses: vakli.pal@ttk.mta.hu (Pál Vakli), meszlenyi.regina@ttk.mta.hu (Regina J. Deák-Meszlényi), hermann.petra@ttk.mta.hu (Petra Hermann), vidnyanszky.zoltan@ttk.mta.hu (Zoltán Vidnyánszky)

Abstract

Background

Deep learning is gaining importance in the prediction of cognitive states and brain pathology based on neuroimaging data. Including multiple hidden layers in artificial neural networks enables unprecedented predictive power; however, the proper training of deep neural networks requires thousands of exemplars. Collecting this amount of data is not feasible in typical neuroimaging experiments. A handy solution to this problem, which has largely fallen outside the scope of deep learning applications in neuroimaging, is to repurpose deep networks that have already been trained on large datasets by fine-tuning them to target datasets/tasks with fewer exemplars. Here we investigated how this method, called transfer learning, can aid age category classification and regression based on brain functional connectivity patterns derived from resting-state functional magnetic resonance imaging. We trained a connectome-convolutional neural network on a larger public dataset and then examined how the knowledge learned can be used effectively to perform these tasks on smaller target datasets collected with a different type of scanner and/or imaging protocol and pre-processing pipeline.

Results

Age classification on the target datasets benefitted from transfer learning. Significant improvement (~9-13% increase in accuracy) was observed when the convolutional layers' weights were initialized based on the values learned on the public dataset and then fine-tuned to the target datasets. Transfer learning also appeared promising in improving the otherwise poor prediction of chronological age.

Conclusions

Transfer learning is a plausible solution to adapt convolutional neural networks to neuroimaging data with few exemplars and different data acquisition and pre-processing protocols.

Keywords

deep learning, transfer learning, convolutional neural networks, resting-state fMRI, brain age prediction

Background

Deep learning, a branch of machine learning that allows multi-layered neural network models to learn representing data at increasing levels of abstraction [1], is gaining importance in the analysis of brain imaging data [2], and has been applied successfully in neuroimaging studies of psychiatric and neurological disorders [3]. As an example, our group has successfully applied deep learning for fMRI-based classification of amnestic mild cognitive impairment [4]. More specifically, we presented a novel convolutional neural network (CNN) architecture that efficiently distinguished between subject groups based on functional connectivity metrics derived from resting-state fMRI measurements.

While these methods have the potential to revolutionize fMRI data analysis [2] and provide a conceptual framework for understanding brain function [5], training deep neural networks comes at a cost. This is mainly because many examples are required to properly train these models. A rough rule of thumb is that to achieve agreeable performance, a supervised deep learning algorithm requires around 5000 labelled training examples per category [6]. Accordingly, datasets used in several areas of machine learning are often enormous. For

example, the AlexNet [7], a CNN model that achieved a breakthrough in natural image recognition in 2012 [8], was trained on roughly 1.2 million examples from the ImageNet database [9]. This is in stark contrast with the sample size in typical neuroimaging experiments. In a recent review of more than 200 studies using neuroimaging and machine learning for the classification of patients with various brain disorders, the authors found that the median sample size of all studies was 88 [10]. By contrast, the number of features (regions or voxels) in neuroimaging experiments is typically far greater—in the field of functional connectomics, it ranges from the order of tens to 1 million [11]. Complex models trained under such circumstances are prone to learn the idiosyncratic details of the sample data instead of the general functional relationship between brain activation patterns and cognitive states. For this reason, such models show poor generalization to samples they have never encountered before, a phenomenon which is commonly referred to as 'overfitting' [12,13].

Open sharing of neuroimaging data is envisaged by many as a possible solution to the problem of small sample sizes [10]. Significant progress has been made in this area, as now there are more than 8000 shared MRI datasets available online [14]. However, data sharing entails the possibility of introducing undesirable variability into data analysis, which is a central issue in multicenter fMRI studies, and is related to differences in scanner types, sequence parameters, stimulus presentation, and image processing between research sites [15]. In addition, the increased computational burden of processing vast amounts of neuroimaging data should also be taken into account [2]. Considering these limitations, the question arises as to how data from different sources can be combined effectively for deep learning applications in neuroimaging.

In machine learning, it is not uncommon to rely on previous knowledge instead of training a model from scratch. Transfer learning [16] refers to the method of training a model on one dataset (the source domain) and then transfer the acquired knowledge—which is, in the case of neural networks, manifest in the learned weights—to train a model on a different dataset and/or

task (the target domain). This method is useful when the source and target datasets differ in terms of feature space or data distribution [16] and can be used effectively when the target dataset is too small to train a large network without overfitting [17]. As a recent example, [18] harnessed the image representations learned by a CNN on a large-scale dataset (the ImageNet; see above) in order to perform various visual recognition tasks on a dataset with only limited amount of training examples. In particular, the pre-trained parameters of the internal layers were transferred to the target task and kept constant, while the last fully connected layer was replaced by two new layers that were trained on the target dataset. This transfer learning method led to enhanced performance when compared to state-of-the-art models, despite differences in image statistics and tasks between the two datasets [18]. Other examples include keeping the weights of the pre-trained layers fixed and training a linear regression or support vector machine (SVM) classifier on top to adapt the model to the target domain [19–21].

Yosinski et al. [17] trained a CNN for visual classification on one dataset and then systematically examined the extent to which transferring parameters from different layers aids the retraining of the remaining layers on a similar dataset. The authors found that the first two layers show almost perfect transfer, in line with the frequently observed phenomenon that when deep neural networks are trained on images, the resulting representations in their first layers—i.e. Gabor filters or color blobs [22]—are general in the sense that they can be applied to many datasets and tasks. Transferring deeper layers, however, led to a significant drop in performance due to the representations being more specific to the source domain as well as due to the loss of co-adapted representations between successive layers. Interestingly, transferring the weights only to initialize the network which is then fine-tuned to the target dataset resulted in better performance than when the network was trained directly on the target dataset. This suggests that transfer learning may be desirable even when the target domain has sufficient examples to train the network without overfitting [17].

Taken together, the above results suggest that transfer learning is beneficial when the sample size in the target domain is too small to train deep neural networks without overfitting. The effectiveness of this method depends on the use of knowledge about the source domain, i.e. which layers are transferred and whether the weights are fixed or used only to initialize the network when training on the target dataset. While these studies focused on how to deal with the scarcity of data in specific natural image recognition tasks, transfer learning has to potential to alleviate the problem of small sample size in neuroimaging.

In the present study, we performed a systematic investigation of how knowledge can be extracted effectively from a model that has already been trained on a publicly available dataset. In particular, we examined how transfer learning can be used to adapt a CNN to a relatively small dataset to predict age from functional neuroimaging data. Predicted brain age is attracting significant attention due to its potential as a biomarker of individual brain health [23], and recent results show that deep learning is effective in predicting age from structural MRI data [24]. In the current study, region-of-interest-based whole-brain resting-state functional connectivity matrices acquired in our own lab from subjects of two age categories (elderly and young) constituted the target domain. The source domain consisted of functional connectivity matrices and corresponding chronological age labels resulting from the aggregation of publicly available data. The two datasets differed markedly in size and data acquisition (scanner type and imaging sequence) and pre-processing parameters. We examined how weights from certain layers of our CNN model trained on the source dataset can be used to enhance chronological age classification and regression performance on the target dataset. We also investigated how the contribution of the connectivity fingerprints of brain regions and networks to classification performance changed in different transfer learning conditions. Finally, the generalizability of the proposed transfer learning method was tested by examining an alternative target dataset derived from publicly available data.

Data description

The source dataset in this study was obtained by aggregating publicly available datasets (hereafter referred to as the public dataset). We used a target dataset that was acquired in our own lab (the in-house dataset). In addition, to test the generalizability of the proposed method, we also examined an alternative target dataset (the NKI-RS subset) consisting of publicly available data. See the Methods section for full details of the data acquisition and pre-processing pipelines.

Public dataset

We used publicly available data from Consortium for Reliability and Reproducibility (CoRR; [25]): the LMU 1, 2 and 3 datasets [26,27]; and from the International Data Sharing Initiative (INDI; [28]): the Southwest University Adult lifespan Dataset (SALD) [29]. The aggregated public dataset includes 368 resting-state fMRI measurements from 200 subjects (117 females) aged between 19 and 30 years (mean ± SD = 23.9 ± 2.4 years; the young age group), 144 measurements from 144 subjects (92 females) aged between 31 and 54 years (mean ± SD = 44.8 ± 6.6 years; the middle-aged group), and 332 measurements from 237 subjects (141 females) aged between 55 and 80 years (mean ± SD = 64.2 ± 6.9 years; the elderly age group). Measurements from the young and elderly age groups were used for classification. Measurements from the middle-aged group were omitted from classification. Measurements from all three groups were used for regression.

In-house dataset

A total of 57 subjects with no history of neurological or psychiatric diseases and normal or corrected-to-normal visual acuity participated in the experiment. 28 subjects (14 females) were aged between 20 and 33 years (mean ± SD = 23.9 ± 2.7 years; the young age group) and 29

subjects (14 females) were aged between 59 and 90 years (mean ± SD = 68.7 ± 6.1 years; the elderly age group). Each subject underwent an anatomical scan and a subsequent 600-sec-long resting-state fMRI measurement. Subjects were instructed to lie still while fixating a dark spot in the centre of the screen on a grey background.

The NKI-RS subset

In addition to the in-house dataset, the proposed method was tested on an alternative target dataset derived from the enhanced Nathan-Kline Institute Rockland Sample (NKI-RS) [30]. Similarly to the in-house dataset, measurements in the NKI-RS dataset were obtained using simultaneous multi-slice imaging, albeit with different parameters and using a different type of scanner (see the Methods section for details). A subset of the participants in the NKI-RS dataset was selected randomly, with the only constraints being that the size of the resulting dataset and the age range of the participants match the in-house dataset as closely as possible. The resulting NKI-RS subset included 30 young subjects (17 females) aged between 20 and 32 years (mean ± SD = 24.4 ± 3.5 years) and 30 elderly subjects (18 females) aged between 59 and 80 years (mean ± SD = 68.2 ± 6.3 years). A single 580.5-sec-long resting-state fMRI measurement was used for each participant.

The limited size of the NKI-RS subset allowed us to investigate the potential benefit of transfer learning when dealing with small samples like the in-house dataset. Moreover, the NKI-RS imaging protocol (simultaneous multi-slice imaging) and the pre-processing pipeline it necessitates (removal of artifacts using independent component analysis) ensure that, similarly to the in-house dataset, the NKI-RS subset differs from the public dataset and hence it is suitable for testing the potential of transfer learning to improve performance in functional connectivity pattern analysis.

Functional connectivity calculation

To calculate ROI-based whole-brain functional connectivity, we used the Harvard-Oxford Atlas included in FSL [31], consisting of 111 anatomical regions of interest (for the full list of ROIs, see Additional file 1.), to obtain 111 meaningful averaged blood-oxygen-level-dependent (BOLD) signals in each measurement. From these 111 time series we calculated full connectivity matrices leading to 111*110/2 = 6105 independent pairwise connectivity features.

Analyses

Classification

We examined whether the classification of age category (young/elderly) based on resting-state functional connectivity data in a relatively small sample (the in-house dataset) can be improved by transferring the knowledge learned on a larger sample (the public dataset). First, we used the in-house dataset for training a connectome-convolutional neural network (CCNN) as well as testing its performance with cross-validation which served as a baseline. Second, we trained the CCNN on the public dataset and used the resulting weights and bias terms either to directly classify the instances in the in-house dataset, or to guide the further training of the network on the in-house dataset. This resulted in 5 different transfer learning conditions (Figure 1.), and the classification performances in these conditions were compared to the baseline, i.e. when the CCNN was trained solely on the in-house dataset. The network architecture and the different training conditions are detailed in the Methods section.

The classification results are summarized in Table 1. Above-chance classification accuracy was observed (84.2%) when the CCNN was trained exclusively on the in-house dataset ($Conv_{Train}Full_{Train}$). When the CCNN was trained on the public dataset and all the resulting weights were used directly to test the model on the in-house dataset ($Conv_{Const}Full_{Const}$), a slight increase in performance was observed (86%, p = 0.5). Thus, while baseline classification performance is encouraging, there is still room for improvement regarding knowledge transfer.

Importing only the convolutional layers' weights and biases and training the fully connected layers from scratch on the in-house dataset ($Conv_{Const}Full_{Train}$) led to a more pronounced improvement in classification performance (91.2%), even though the difference to the baseline condition ($Conv_{Train}Full_{Train}$) did not reach the level of significance ($p = 0.172$). When the weights of the fully connected layers were initialized based on the values learned on the public dataset ($Conv_{Const}Full_{Init}$), a similar result was obtained (91.2%; $p = 0.172$). Finally, initializing the weights of the convolutional layers based on previously learned values led to a significant improvement in classification performance over the baseline condition (93% and $p = 0.031$ for both $Conv_{Init}Full_{Train}$ and $Conv_{Init}Full_{Init}$). On the whole, training the CCNN on both datasets consistently led to better results than when the model was trained exclusively on one dataset, and a significant improvement was observed when the convolutional layers were fine-tuned on the target dataset after learning from the source dataset.

Importantly, when the weights learned on the in-house dataset were used to classify instances in the public dataset (Back-transfer), performance dropped dramatically (60.9%). As the CCNN can classify instances of the in-house dataset in the $Conv_{Train}Full_{Train}$ condition with 84.2% accuracy (with cross-validation), we can claim that the CCNN does not simply overfit the small dataset. The poor generalization of these representations to the public dataset suggests, however, that the CCNN probably learned specific details of the in-house dataset. That is, it relies on connectivity differences between subject groups that are enlarged due to the parameters of our measurements, like the very short TR due to multi-slice imaging. In contrast, the representations learned on a larger and more diverse dataset generalize well to the in-house data, as the classification accuracy in the $Conv_{Const}Full_{Const}$ condition was 86%. This shows that the connectivity differences the CCNN relies on in this case are substantial regardless of scanner type and imaging parameters.

ROIs relevant for classification

We investigated the role of the connectivity patterns of individual brain regions and large-scale brain networks in the decision-making process when the CCNN was trained on the public dataset ($Conv_{Const}Full_{Const}$), on the in-house dataset ($Conv_{Train}Full_{Train}$), or when it was fine-tuned to the in-house dataset after training on the public dataset ($Conv_{Init}Full_{Train}$ and $Conv_{Init}Full_{Init}$). To this end, we performed an occlusion test (see the Methods section for details). Briefly, we replaced the connectivity fingerprints of individual ROIs or groups of ROIs constituting a given brain network with zeros in the input and re-classified the instances in the in-house dataset, with weights and bias terms as constants corresponding to the values established at the end of the training process in the given condition. The most important brain regions/brain networks were considered to be the ones the occlusion of which resulted in a substantial drop in classification accuracy.

Classification performance did not change considerably when the connectivity fingerprints of individual ROIs were occluded (Additional files 2. and 3.). The only notable effect was a ~9% drop in accuracy when the anterior division of the right supramarginal gyrus was occluded in the $Conv_{Const}Full_{Const}$ condition. Otherwise, the mean accuracy change was -0.3% (SD = 1%) across conditions.

Classification performance proved to be more sensitive to the occlusion of large-scale brain networks. When trained solely on the in-house dataset ($Conv_{Train}Full_{Train}$), the CCNN seemed to rely heavily on the connectivity pattern of the default mode network, whose occlusion resulted in a 12.3% drop in accuracy (Figure 2. right panel). In contrast, when the network was trained on the public dataset only ($Conv_{Const}Full_{Const}$), the regions of the visuospatial network turned out to be crucial for classification, as the occlusion of these regions resulted in below-chance performance (56.1%; Figure 2. left panel). The visuospatial network remained highly important when the CCNN was fine-tuned to the in-house dataset, along with the executive control network in the $Conv_{Init}Full_{Init}$ condition (8.8% drop in accuracy; Figure 3. left panel)

and the sensorimotor network in the $Conv_{Init}Full_{Train}$ condition (10.5% drop in accuracy; Figure 3. right panel). The regions corresponding to these networks are shown in Figure 4.

Regression

We modified the CCNN model to regress chronological age against functional connectivity patterns (see Methods section for details). When the CCNN was trained solely on the in-house dataset to regress chronological age with the ROIs' functional connectivity fingerprints as independent variables, performance was rather poor (MAE = 12.55 years, Pearson's r = 0.75, R^2 = 0.5, RMSE = 16.09 years). Using the convolutional layer weights learned on the public dataset for age category classification and then fine-tuning the fully connected layers to perform regression on the in-house data, however, resulted in a remarkable improvement in regression performance (MAE = 7.77 years, Pearson's r = 0.84, R^2 = 0.71, RMSE = 12.39 years; Figure 5.). The difference in performance between the baseline and transfer learning conditions was significant ($t = 3.46$, $p = 0.001$).

The NKI-RS subset

To assess the generalizability of the transfer learning findings obtained using the in-house dataset, the same transfer learning conditions were also tested using the NKI-RS subset as the target dataset. By and large, transferring the knowledge learned on the public dataset to aid classification performance on the NKI-RS yielded a pattern of results that was comparable to the one observed in the case of the in-house dataset. When the CCNN was trained exclusively on the NKI-RS subset ($Conv_{Train}Full_{Train}$), classification accuracy was 75%. Only a modest increase in performance was observed (76.7%, p = 0.5) when the CCNN was trained on the public dataset and all the resulting weights were used directly to classify instances in the NKI-RS subset ($Conv_{Const}Full_{Const}$). Importing only the convolutional layers' weights and biases and keeping them constant lead to a greater increase in performance, although the difference to the

baseline condition ($Conv_{Train}Full_{Train}$) did not reach the level of significance ($Conv_{Const}Full_{Train}$: 85%, p = 0.073; $Conv_{Const}Full_{Init}$: 81.7%, p = 0.212). Finally, initializing the weights and biases of the convolutional and fully connected layers based on previously learned values ($Conv_{Init}Full_{Init}$) led to a significant improvement in classification performance over the baseline condition (88.3%, p = 0.004). This suggests that fine-tuning the convolutional layers after learning from the public dataset is important for improving classification performance significantly, similarly to the in-house dataset. Initializing only the convolutional layers' weights and biases ($Conv_{Init}Full_{Train}$) also increased performance, although this increase did not reach the level of statistical significance (85%, p = 0.055).

Examining the role of large-scale brain networks in classification using the occlusion test revealed that transfer learning had a similar effect to the one observed in the case of the in-house dataset. The CCNN relied heavily on the default mode and visuospatial networks (8.33% drop in accuracy) when trained exclusively on the public dataset ($Conv_{Const}Full_{Const}$; Additional figure 5. left panel), and on the visuospatial and salience networks (1.67% drop in accuracy) when trained solely on the NKI-RS subset ($Conv_{Train}Full_{Train}$; Additional figure 5. right panel). When the CCNN was fine-tuned to the NKI-RS subset ($Conv_{Init}Full_{Init}$ and $Conv_{Init}Full_{Train}$; Additional figure 6.) the default mode network became the most important set of brain regions for classification among the ones that were tested (8.3 and 13.33% drop in accuracy, respectively).

As of chronological age regression, performance was extremely poor when the CCNN was trained solely on the NKI-RS subset (MAE = 14.97 years, Pearson's r = 0.6, R^2 = 0.26, RMSE = 19.95 years). Transfer learning improved regression performance (MAE = 12.25 years, Pearson's r = 0.7, R^2 = 0.46, RMSE = 17.08 years), albeit the difference between the baseline and transfer learning conditions did not reach significance (t = *1.53*, p = 0.13).

Discussion

In the present study, we trained a connectome-convolutional neural network to perform binary chronological age category classification (young/elderly) based on region-of-interest-based resting-state functional connectivity patterns derived from fMRI measurements. Even though baseline classification was well above chance, we found that performance could be improved further by training the CCNN model on a larger, publicly available dataset and then making use of the knowledge learned to classify instances in the smaller in-house dataset. This has occurred despite the fact that the two datasets differed considerably in terms of the data acquisition protocol (scanner type and imaging sequence) and pre-processing parameters. A similar result was obtained when the knowledge learned on the public dataset was transferred to the NKI-RS subset, suggesting that transfer learning is generally applicable for the augmentation of functional connectivity pattern classification.

Applying the model trained on the public dataset one-in-one to the in-house dataset or NKI-RS subset resulted in a modest ~2% increase in performance compared to the baseline condition, i.e. when the model was trained exclusively on either of the target datasets. This suggests that the representations learned on the public dataset are rather abstract and generalize to other datasets reasonably well—nevertheless, classification performance could benefit from continued learning on the target dataset as well. Indeed, a significant improvement in classification performance was observed when the convolutional layers' weights were initialized on the basis of the previously learned values and then trained on the in-house dataset or the NKI-RS subset. In general, these results suggest that a handy solution to repurpose existing convolutional neural network models for functional connectivity pattern classification is to fine-tune the convolutional (as well as the fully connected) layers to the target dataset by initializing the weights with the previously learned values. This outcome bears a close

resemblance with previous results in the field of natural image recognition. In particular, [17] found that an eight-layer convolutional neural network trained on a large source image dataset and then fine-tuned to a target dataset shows better generalization that those trained directly on a target dataset of the same size. The authors came to the conclusion that the initialization of network weights with transferred values might be a generally useful method for improving CNN performance, even when the target dataset is large enough to train the network from scratch without overfitting.

Deep learning is a highly promising method for inferring cognitive states and brain pathology from neuroimaging data [2]. In particular, convolutional neural networks have been applied successfully to make predictions on the basis of brain structure [24] and functional connectivity patterns [4]. However, a major drawback of these networks is that their proper training requires extensive amounts of data [6], which substantially exceeds the sample sizes in typical neuroimaging experiments [10]. Performing neuroimaging measurements in the order of thousands to train deep networks from scratch to answer specific research questions under specific data acquisition and processing protocols is impracticable. Nevertheless, with the advent of neuroimaging 'big data' [14], reusing models that have been trained on large-scale datasets seems to be a viable solution to tackle the 'data-hungry' nature of CNNs. This situation is comparable to that in natural image recognition, where large-scale annotated image sets are available (e.g. [9]), and the weights of CNNs trained on such datasets can be transferred effectively to solve visual recognition tasks with limited training data (e.g. [18]). There have been several attempts lately that combined auxiliary datasets for the classification of brain disease states in SVM [32,33] or multinomial regression [34] settings effectively. Recently, Mensch et al. [35] used several datasets from different brain imaging studies simultaneously to train a shared multi-layered architecture to decode cognitive states from neural activity patterns. The authors found that aggregating multiple datasets boosted decoding performance

on a target dataset, and this gain in accuracy increased with smaller training size. This offers the potential of learning representations of neural activity from already existing data repositories that can be generalized to newly acquired fMRI data [35]. Our results suggest that transfer learning might be a useful method in applying deep neural networks that have already been trained on larger datasets to fMRI data with a limited number of exemplars. More specifically, the results of the present study also imply that the fine-tuning of convolutional layers by weight initialization is a handy solution to adapt a CNN to the target dataset, at least in the domain of functional connectivity pattern classification.

It is important to note that even though baseline classification performance was well above chance, when the model trained on the in-house dataset was used directly to classify instances in the public dataset, performance dropped to below chance level. This is indicative of a special type of overfitting [12,13]; the CCNN probably learned the idiosyncratic details of the in-house dataset, that correlate with age, but came from our specific measurement parameters instead of the general relationship between functional connectivity patterns and chronological age. This implies that even remarkably good performance should be treated with caution when deep networks are trained on small datasets, and transfer learning might be beneficial under such circumstances as well.

We also investigated which brain region's connectivity patterns played an important role in age category classification of the in-house and NKI-RS exemplars in the different training conditions by examining changes in classification performance resulting from the occlusion of each region's connectivity fingerprint in the input. It turned out that classification accuracy was largely unaffected by the occlusion of individual brain regions. This insensitivity to slight changes in the input might be due to the use of dropout (with a dropout rate of 0.4) which increased the robustness of the CCNN to the occlusion of features. However, removing the connectivity patterns of large-scale brain networks from the input had a greater effect on

classification performance. It appeared that the CCNN relied on the connectivity patterns of different brain networks when trained either on the target or public dataset. This latter set of brain regions tended to play a dominant role in the classification of target dataset exemplars when the CCNN was fine-tuned to the target dataset after training on public data. Thus, it seems likely that fine-tuning the network to the target dataset entailed the involvement of a combination of brain regions that is more generally related to the aging process.

Besides age category classification, we also trained our CCNN model to predict chronological age based on brain functional connectivity patterns. When the network was trained exclusively on the in-house dataset, age regression performance was rather poor. Nonetheless, the application of transfer learning lead to a significant improvement. In particular, keeping the convolutional layer weights that were used successfully in categorization and fine-tuning the fully connected layers to the in-house dataset for the purpose of regression enabled a more accurate prediction of chronological age. While transfer learning also improved age prediction in the case of the NKI-RS subset, this improvement did not reach the level of significance—this might be due to the extremely poor baseline performance which transfer learning could not overcome.

Evidence is mounting that brain age—the predicted age of an individual that is derived from neuroimaging data—is related to physical health and brain disease [23]. As such, it is a promising biomarker for individual brain health. Recently, Cole et al. [24] predicted chronological age with less than five years mean absolute error using a CNN trained on T1-weighted structural MRI scans from 1601 healthy individuals. The authors found that even though the within-scanner reliability of brain-predicted age was high, between-scanner reliability was markedly reduced, especially for T1 scans with minimal pre-processing. Thus, multi-center reliability seems to be an issue for CNN-based age estimation, at least when raw structural MRI scans are used for prediction. The precision of age estimation in our study

remained well below the one reported by Cole et al. [24]. Regression performance in the transfer learning condition would certainly have benefitted from a larger dataset— the public dataset in our study was approximately half the size of the one used by Cole et al. [24]. Nevertheless, our results suggest that transfer learning is a potentially useful method to enhance the prediction of chronological age based on resting-state brain functional connectivity pattern analysis. In particular, weight transfer in the convolutional layers and fine-tuning of the fully connected layers to the target dataset seem to represent a promising solution to adapt CNNs to data acquired with different scanner types and imaging protocols for the purpose of predicting brain age. The effectiveness of this method shall be investigated further with possibly larger source datasets.

Potential implications

We believe that transfer learning has the potential to alleviate the problem of data scarcity regarding deep learning applications in neuroimaging. Here we showed that an already-trained CNN can be fine-tuned effectively to a fMRI functional connectivity dataset with different data acquisition and pre-processing parameters. Since the initial convolutional layers in CNNs tend to learn more general representations [17], it is plausible that models trained on large datasets can also be repurposed to perform a variety of different tasks at relatively low cost.

Methods

Public data acquisition and pre-processing

The LMU 1, 2, and 3 datasets were collected at the Institute of Clinical Radiology, Ludwig-Maximilians-University, Munich, Germany. Data from the LMU 1 dataset were acquired using a Philips Achieva 3T MRI scanner (Best, The Netherlands) and a 32-channel headcoil. High-resolution anatomical images were acquired for each of the 26 subjects (14 females; age (mean

± SD) = 24.3 ± 1.9 years) using a T1-weighted 3D TFE sequence (1 mm isotropic voxels; slice thickness/slice gap = 1/0 mm; repetition time (TR) = 2375 ms; flip angle (FA) = 8°; field of view (FOV) = 240 mm; acceleration factor = 2/2.5). A total of 180 functional images over 455 secs were collected with a BOLD-sensitive T2*-weighted gradient-echo-EPI sequence (slice thickness/slice gap = 3/0 mm; slice in-plane resolution = 2.95 × 2.95 mm; TR = 2500 ms; echo time (TE) = 30 ms; FA = 90°; FOV = 224 × 233 mm; acceleration factor = 3). Fifty-two axial slices were acquired in ascending acquisition order covering the whole brain. Each subject participated in at least five 455-sec-long resting-state fMRI measurements. Further details are available on the website of the dataset [36].

Data from the LMU 2 and 3 datasets were acquired using Siemens Magnetom Verio and TrioTim 3T MRI scanners (Siemens, Erlangen, Germany), respectively, and 12-channel headcoils. High-resolution T1-weighted anatomical images were acquired for each of the 65 subjects (31 females; age (mean ± SD) = 58.1 ± 20.4 years) using a 3D magnetization-prepared rapid gradient echo (MPRAGE) sequence and 2-fold GRAPPA acceleration with a Partial Fourier factor of 7/8 (1 mm isotropic voxels; slice thickness/slice gap = 1/0.5 mm; TR = 2400 ms; TE = 3.06 ms; FA = 9°; FOV = 256 ms). A total of 120 functional images over 366 secs were collected with a BOLD-sensitive T2∗ weighted gradient-echo EPI sequence (slice thickness/slice gap = 4/0.4 mm; slice in-plane resolution = 3 × 3 mm; TR = 3000 ms; TE = 30 ms; FA = 80°; FOV = 192 mm). Twenty-eight and thirty-six axial slices were acquired in ascending order for the LMU2 and 3 datasets, respectively. In the LMU 2 dataset, each subject participated in four 366-sec-long resting-state fMRI measurements. In the LMU 3 dataset, each subject participated in two 366-sec-long resting-state fMRI measurements. Further details are available on the websites of the datasets [37,38].

The Southwest University Adult lifespan Dataset (SALD) was collected at the Southwest University Center for Brain imaging using a Siemens Magnetom TrioTim 3T MRI scanner (Siemens Medical, Erlanger, Germany). High resolution T1-weighted anatomical images were acquired for 493 subjects (306 females; age (mean ± SD) = 45.2 ± 17 years; one subject lacked functional images and therefore was omitted from the analysis) using an MPRAGE sequence and 2-fold GRAPPA acceleration (TR = 1900 ms; TE = 2.52 ms; FA = 9°; FOV = 256 mm; 1 mm isotropic spatial resolution). A total of 242 functional images over 488 secs were collected using a gradient-echo-EPI sequence (32 slices; slice thickness/slice gap = 3/1 mm; TR = 2000 ms; TE = 30 ms; FA = 90°; FOV = 220 mm; voxel size = 3.4 × 3.4 × 3 mm). Each subject participated in one 488-sec-long resting-state fMRI measurement. Further details are available on the website of the dataset [39].

The public dataset resulted from the aggregation of the LMU 1, 2, 3, and SALD datasets. Pre-processing of the imaging data was performed using the SPM12 toolbox [40] and custom-made scripts running on MATLAB 2015a (The MathWorks Inc., Natick, MA, USA). Each subject's functional images were motion-corrected, the T2*-weighted functional images in all sessions were spatially realigned to the first volume. Then, the realigned functional images were spatially smoothed using a 5 mm full-width half maximum Gaussian filter. The T1-weighted anatomical images in each session were coregistered to the mean T2*-weighted functional images created during the realignment step. The coregistered anatomical images were segmented using the unified segmentation and normalization tool of SPM12. The resulting grey matter (GM) mask was later used to restrict the analysis of the functional images to GM voxels; while the white matter (WM) and cerebrospinal fluid (CSF) masks were used to extract nuisance signals that are unlikely to reflect neural activity in resting-state time-series. The realigned functional images were normalized to the MNI-152 space using deformation field parameters generated during the segmentation and normalization of the anatomical images.

After regressing out the head-motion parameters, the mean WM, CSF, and whole-brain signals [41], residual time courses from all GM voxels were band-pass filtered using a combination of temporal high-pass (based on the regression of ninth-order discrete cosine transform basis set) and low-pass (bidirectional 12th-order Butterworth IIR) filters to retain signals only within the range of 0.009 and 0.08 Hz [42].

In-house data acquisition and pre-processing

Data were acquired on a Siemens Magnetom Prisma 3T MRI scanner (Siemens Healthcare, Erlangen, Germany) at the Brain Imaging Centre, Research Centre for Natural Sciences, Hungarian Academy of Sciences. All head elements of the standard Siemens 64-channel head-neck receiver coil were applied. The protocol consisted of T1-weighted 3D MPRAGE anatomical imaging using 2-fold in-plane GRAPPA acceleration (TR/ TE/ FA = 2300ms/3ms/9°; FOV = 256 mm; isotropic 1 mm spatial resolution). A blipped-CAIPI simultaneous multi-slice (SMS) gradient-echo-EPI sequence [43] was used for functional measurements with 6-fold slice acceleration, using full brain coverage with an isotropic 2 mm spatial resolution and a TR of 710 ms, without in-plane parallel imaging. A Partial Fourier factor of 7/8 was used to achieve a TE of 30 ms. Image reconstruction was performed using the Slice-GRAPPA algorithm [43] with LeakBlock kernel [44].

Pre-processing of the imaging data was performed using SPM12 [40] and FSL 5.0.9 [45] toolboxes as well as custom-made scripts running on MATLAB 2015a (The MathWorks Inc., Natick, MA, USA). The T2*-weighted functional images were spatially realigned to the first volume for motion correction and coregistered with the T1-weighted anatomical image which was then segmented and normalized to the MNI-152 space using the unified segmentation-normalization tool of SPM12. The resulting GM mask was later used to restrict the analysis of the functional images to GM voxels; while the WM and CSF masks were used to extract

nuisance signals that are unlikely to reflect neural activity in resting-state time-series. On the realigned and coregistered functional images spatial Independent Component Analysis using FSL's MELODIC ICA 3.14 [46] was performed at the single subject-level to remove artefacts due to an interaction of the multi-slice acquisition with head motion [47].

After the ICA-based cleaning procedure, functional images were normalized to MNI-152 space using deformation field parameters acquired during the segmentation and normalization of the anatomical image, followed by a 5-mm isotropic Gaussian smoothing. After regressing out the head-motion parameters, the mean WM and CSF signals [41], residual time courses from all GM voxels were band-pass filtered using a combination of temporal high-pass (based on the regression of ninth-order discrete cosine transform basis set) and low-pass (bidirectional 12th-order Butterworth IIR) filters to retain signals only within the range of 0.009 and 0.08 Hz [42].

Acquisition and pre-processing of the NKI-RS subset

MRI data from the enhanced Nathan-Kline Institute Rockland Sample (NKI-RS) [30] were collected using a Siemens Magnetom Trio Tim 3T MRI scanner_(Siemens Healthcare, Erlangen, Germany). High resolution T1-weighted anatomical images were acquired using an MPRAGE sequence and 2-fold GRAPPA acceleration (TR = 1900 ms; TE = 2.52 ms; FA = 9°; FOV = 250 mm; 1 mm isotropic spatial resolution). One 580.5-sec-long resting state fMRI measurement with a total of 900 functional images was used for each individual in the randomly selected subset of participants. A blipped-controlled-aliasing multiband EPI sequence [48] was used for functional measurements with 4-fold slice acceleration (40 slices; TR = 645 ms; TE = 30 ms; FA = 60°; FOV = 222 mm; voxel size = 3.0 × 3.0 × 3.0 mm). Further details are available on the website of the dataset [49]. These measurements were processed in the same way as those in the in-house dataset.

Connectome-convolutional neural network architecture

We used a slightly modified version of our connectome-convolutional neural network model that has previously proved successful in the classification of functional connectivity patterns [4]. In detail, we arranged the connectivity features into 111 × 111 matrices (corresponding to the 111 ROIs) and applied line-by-line convolution (filter size: 1 × 111) followed by convolution by column (filter size: 111 × 1). Thus, we treated the connectivity fingerprint of each ROI (rows in the input matrix) as a unit whose weights can be shared across the whole connectivity matrix. This is based on the assumption that the learned convolutional filter will assign large weights to ROIs that show altered connectivity between the age groups, and thus connectivity strength with those altered regions will have a large influence on the output [4].

In the first convolutional layer we trained 64 filters, i.e. 64 differently weighted sums of each ROI's connectivity fingerprint were calculated. In the second convolutional layer we trained 256 filters. The output of this layer is fed into a fully connected hidden layer with 96 neurons that are connected to the output layer consisting of two neurons corresponding to the two classes. We applied rectified linear unit (ReLU; [50]) non-linearity in the convolutional neural network and the softmax function [51] on the output layer to calculate the probability of each instance belonging to a certain class. The network is trained with cross-entropy as a loss function [6]. To train a robust classifier, we applied dropout regularization [52,53] with keep probability of 0.6 and an Adam optimizer [54] with a learning rate of 0.001 and 5000 training iterations. The two convolutional layers of the CCNN model include 111*64+111*64*256 = 1825728 trainable weights and 320 bias terms. The fully connected hidden and output layers include 256*96+96*2 = 24768 trainable weights and 98 bias terms.

The CCNN model was implemented in Python using TensorFlow 1.3. We used a single NVIDIA Quadro M4000 GPU to train the CCNN. Training on the public dataset for classification took 112 secs. Training on the in-house dataset with 10-fold cross-validation

($Conv_{Train}Full_{Train}$) took 1127 secs. The computation times in the classification transfer learning conditions for the in-house dataset were as follows: 104 secs in $Conv_{Const}FUll_{Train}$, 92 secs in $Conv_{Const}Full_{Init}$, 1163 secs in $Conv_{Init}Full_{Train}$, and 1104 secs in $Conv_{Init}Full_{Init}$. Training and evaluation in the baseline and transfer learning regression conditions took 3417 and 474 secs, respectively.

Transfer learning for classification

To establish the baseline classification performance (i.e. when the in-house dataset is used for both training and testing), we applied cross-validation. Measurements from the 57 subjects were randomly divided into 10 folds. Measurements in each fold constituted the test set for that fold with five or six subjects while the remaining measurements constituted the training set. This same partitioning was used to evaluate all classifiers and conditions. As in this case, the convolutional as well as the fully connected layers were trained in each fold, we refer to this condition as $Conv_{Train}Full_{Train}$.

To evaluate the transfer of weights and bias terms from the public to the in-house dataset, the CCNN was trained on all instances of the public dataset in one fold. Measurements in the in-house dataset were omitted from training. Subsequently, weights and bias terms learned on the public dataset were transferred to classify instances in the in-house dataset. In one condition, the resulting weights and bias terms of the convolutional as well as the fully connected layers were used to classify each instance in the in-house dataset. Since all layers' weights and biases terms were constants based on what had been learned on the public dataset previously, we refer to this condition as $Conv_{Const}Full_{Const}$. In another condition, after training the CCNN on all instances of the public dataset, the model was further trained and evaluated on the in-house dataset using the 10-fold cross-validation scheme. At this stage, the weights and bias terms of the two convolutional layers were kept constant while those of the fully connected layers were

newly initialized (using ‘Xavier’ initialization; [55]) and trained in each fold of the cross-validation (*Conv*$_{Const}$*Full*$_{Train}$).

To examine whether the representations learned on the smaller in-house dataset can be generalized to the larger dataset, we transferred weights and biases learned on the in-house dataset to classify instances in the public dataset. We refer to this condition as *Back-transfer*, as in this case, the direction of knowledge transfer is the opposite to that in the rest of the conditions. In particular, this condition is the exact opposite of the *Conv*$_{Const}$*Full*$_{Const}$ condition, inasmuch as the weights of the convolutional and fully connected layers learned on the in-house dataset are used as constants when testing on the public dataset. Performance in this condition is supposed indicate whether the representations learned on such a small dataset are general in the sense that they concern the relationship between functional connectivity and age, or specific to the characteristic features of the in-house dataset.

We also examined the effect of weight initialization based on the public dataset. Similarly to the previous ones, the conditions described hereinafter involve the training of the CCNN on all instances of the public dataset as a first step. In the second step, instead of keeping the weights and bias terms learned on the public dataset constant, their values are used to re-initialize the same weights and bias terms for training on the in-house dataset in each fold of the cross-validation. In one condition, all layers’ weights and bias terms were initialized based on what had been learned previously on the public dataset (*Conv*$_{Init}$*Full*$_{Init}$). In a further condition, only the convolutional layers’ parameters were initialized based on the previously learned values, while the weights and biases of the fully connected layers were newly initialized in each fold using ‘Xavier’ initialization (*Conv*$_{Init}$*Full*$_{Train}$). Finally, we examined performance in a condition in which the weights and biases of the convolutional layers were kept constant while those of the fully connected layers were initialized using the values learned on the public dataset (*Conv*$_{Const}$*Full*$_{Init}$).

We assessed classification performance with two metrics: accuracy (the proportion of correctly classified instances) and area under the receiver operating characteristic curve (AUC).

To determine the baseline of random classification, we applied a binomial method [13]. In two class classification, the random classifier has p = 50% chance of predicting the true label, therefore the probability of obtaining not more than k correct labels out of n trials can be calculated from the cumulative binomial distribution function:

$$F_{Binom}(n,k,p) \quad = \sum_{i=0}^{k} \binom{n}{i} p^{i}(1-p)^{n-i}$$

For the threshold of significance we chose the 95th percentile, i.e., we searched for the k value where $F_{Binom}(n, k, 0.5) \approx 0.95$. From this, the baseline accuracy can be calculated as k/n. In the case of the in-house dataset, the calculated baseline accuracy is 63.16% with $F_{Binom}(57, 36, 0.5) = 0.969$.

Classification performance in each transfer learning condition was compared separately to the performance obtained in the baseline condition ($Conv_{Train}Full_{Train}$) using a binomial test [56]. In particular, we computed the probability that the transfer model correctly classifies an example that the baseline model misclassifies at least as many times as observed in the experiment, given the assumption that the two models perform equally well, using the formula

$$\sum_{s}^{n} \frac{n!}{s!\,(n-s)!} 0.5^{n}$$

where n is the number of examples for which the two models gave different predictions and s is the number of cases in which the transfer model gave a correct prediction and the baseline model gave an incorrect one. We consider the difference significant if the calculated p-value is lower than 0.05.

Evaluating the role of ROIs in classification

To investigate how the connectivity fingerprints of individual brain regions and large-scale brain networks contribute to classification performance, we systematically occluded different parts of the input correlation matrix and examined how performance changes as a result, in each condition separately. At this stage, the weights and bias terms of the CCNN corresponded to the values learned in the given condition and were kept constant during testing. For example, when evaluating the importance of ROIs in the $Conv_{Const}Full_{Const}$ condition, the weights and biases of the model corresponded to those learned on the public dataset. A similar approach was adopted previously in a study where the authors systematically occluded different parts of a natural input image and monitored the output of the CNN [21].

First, we investigated the role of each anatomical ROI in classification. To this end, we occluded the connectivity fingerprint of the given ROI by setting all the values in the corresponding row and column of the correlation matrix to zero. We then classified each partially occluded correlation matrix in the in-house dataset and examined classification accuracy. We repeated this process for each ROI in the $Conv_{Const}Full_{Const}$, $Conv_{Train}Full_{Train}$, $Conv_{Init}Full_{Init}$, and $Conv_{Init}Full_{Train}$ conditions separately. The full list of anatomical ROIs is shown in Additional file 1. It was considered that the occlusion of the connectivity patterns of brain regions that are crucial for classification would result in a substantial drop in accuracy. Classification accuracies resulting from the occlusion of each region's connectivity fingerprint are displayed in Additional files 2. and 3.

Second, we examined the contribution of networks of brain regions to classification. This approach was based on the atlases of functional ROIs created by Shirer et al. [57]. The authors identified 90 functional ROIs across 14 large-scale brain networks by applying independent component analysis to group-level resting-state data. Here, we mapped these functional ROIs

onto the anatomical ROIs in the Harvard-Oxford Atlas by visual inspection to define the corresponding brain networks. These networks, the constituent anatomical ROIs, and their functional counterparts are listed in Additional file 4. To investigate the role of these networks in age category classification, we occluded the connectivity fingerprints of all ROIs constituting the given network (by setting all the values in the corresponding rows and columns of the correlation matrix to zero; see above) and examined the resulting change in performance. We repeated this process for each network and condition separately. Some of these networks were unified pairwise prior to the occlusion test: the dorsal and ventral default mode networks; the primary and higher visual networks; the anterior and posterior salience networks; and the left and right executive control networks. It was considered that the occlusion of the connectivity patterns of networks of brain regions that are crucial for classification would result in a substantial drop in accuracy. These brain networks are displayed in representative sections of the MNI brain template (Figure 4.). Classification accuracies resulting from the occlusion of each network's connectivity fingerprints are displayed in Figure 2. and 3.

Transfer learning for regression

The CCNN was modified to implement a regression model with the functional connectivity fingerprints of ROIs as independent variables and chronological age as the dependent variable. To this end, the number of neurons in the output layer was reduced to one. The total number of trainable weights in the fully connected layers changed accordingly to 24672 plus 97 bias terms. To train the network, we used mean squared error as the loss function and Adam optimizer with a learning rate of 0.0005 and 15000 training iterations. Dropout regularization with a keep probability of 0.6 was applied. Baseline regression performance was established using the in-house dataset and a 10-fold cross-validation scheme.

To examine how transfer learning from the public dataset aids regression when using the in-house data, the convolutional layer weights and bias terms that had been learned previously on the public dataset to perform binary age category classification were used as constants. First, the fully connected layers of the CCNN were trained on the public dataset to regress chronological age. Second, the fully connected layers were trained to perform regression on the in-house dataset. In this second step, the weights and biases of the fully connected layers were initialized with the values learned on the public dataset in the previous step. Regression performance on the in-house dataset was established using the 10-fold cross-validation scheme.

We evaluated regression performance using the mean absolute error (MAE), the correlation between chronological and predicted age (Pearson's r), the coefficient of determination (R^2), and the root mean squared error (RMSE). We compared the prediction errors (the absolute value of the difference between the true age and the predicted age in years for each exemplar) between the baseline and transfer learning conditions using a paired t-test.

The NKI-RS subset

Transfer learning for age category classification, chronological age regression, and the brain network occlusion test for the NKI-RS subset was implemented in the same way as when the in-house dataset was targeted.

Availability of supporting data and materials

The T1-weighted and T2*-weighted MRI scans, connectivity matrices, and labels are available in the GigaScience repository GigaDB.

Availability of source code and requirements

Project name: Transfer learning for CCNN-based resting-state functional connectivity pattern analysis

Project home page: https://github.com/vaklip/transfer_learning_ccnn

Operating system(s): Platform independent

Programming language: Python

Other requirements: TensorFlow 1.3

License: MIT

The codes used for the pre-processing of the imaging data are available in a separate repository:

Project home page: https://github.com/vaklip/rsfmri_fconn

Operating system(s): Platform independent

Programming language: MATLAB

Other requirements: SPM 12; Tools for NIfTI and ANALYZE image

License: MIT

Additional files

Additional file 1. The list of regions of interest in the Harvard-Oxford Atlas used to calculate ROI-based whole-brain functional connectivity.

Additional file 2. ROI occlusion test results for the $Conv_{Const}Full_{Const}$ and $Conv_{Train}Full_{Train}$ conditions performed on the in-house dataset. The percentage of correctly classified exemplars in the in-house dataset (horizontal axes) are plotted for each occluded ROI (vertical axis). See Additional file 1. for the identification number of each region of interest. Black dashed lines show the classification accuracies in the corresponding conditions when no ROI was occluded. Red dashed lines show the accuracy level corresponding to random classification.

Additional file 3. ROI occlusion test results for the $Conv_{Init}Full_{Init}$ and $Conv_{Init}Full_{Train}$ conditions performed on the in-house dataset. The percentage of correctly classified exemplars in the in-house dataset (horizontal axes) are plotted for each occluded ROI (vertical axis). See Additional file 1. for the identification number of each region of interest. Black dashed lines show the classification accuracies in the corresponding conditions when no ROI was occluded. Red dashed lines show the accuracy level corresponding to random classification.

Additional file 4. Brain networks examined in the occlusion test performed on the in-house and NKI-RS dataset. The functional networks (left column) were defined by Shirer et al. [57]. The constituent functional ROIs (middle column) and their anatomical counterparts in the Harvard-Oxford Atlas (right column) are listed for each network. Anatomical ROIs corresponding to a given functional network were occluded to investigate the contribution of that particular network to age category classification. Note that some of these networks were unified pairwise prior to the occlusion test: the dorsal and ventral default mode networks; the primary and higher visual networks; the anterior and posterior salience networks; and the left and right executive control networks.

Additional file 5. Network occlusion test results for the $Conv_{Const}Full_{Const}$ and $Conv_{Train}Full_{Train}$ conditions performed on the NKI-RS subset. The percentage of correctly classified exemplars in the NKI-RS subset (horizontal axes) are plotted for each occluded functional network

(vertical axes). Black dashed lines show the classification accuracies in the corresponding conditions when no network was occluded. Red dashed lines show the accuracy level corresponding to random classification.

Additional file 6. Network occlusion test results for the $Conv_{Init}Full_{Init}$ and $Conv_{Init}Full_{Train}$ conditions performed on the NKI-RS subset. The percentage of correctly classified exemplars in the in- NKI-RS subset (horizontal axes) are plotted for each occluded functional network (vertical axes). Black dashed lines show the classification accuracies in the corresponding conditions when no network was occluded. Red dashed lines show the accuracy level corresponding to random classification.

DeclarationsList of abbreviations

AUC: area under the receiver operating characteristic curve; BOLD: blood-oxygen-level-dependent; CAIPI: controlled aliasing in parallel imaging; CCNN: connectome-convolutional neural network; CNN: convolutional neural network; CSF: cerebrospinal fluid; EPI: echo-planar imaging; FA: flip angle; fMRI: functional magnetic resonance imaging; FOV: field of view; GM: grey matter; GRAPPA: generalized autocalibrating partial parallel acquisition; NKI-RS: enhanced Nathan-Kline Rockland Sample; ReLU: rectified linear unit; ROI: region of interest; RMSE: root mean squared error; SVM: support vector machine; TE: echo time; TR: repetition time; WM: white matter

Ethics, consent and permissions

Participants gave informed written consent in accordance with the protocols approved by Health Registration and Training Center (ENKK 006641/2016/OTIG), Budapest, Hungary.

Competing interests

The authors declare that they have no competing interests.

Funding

This work was supported by a grant from the Hungarian Brain Research Program (KTIA_13_NAP–A–I/18) to ZV.

Authors' contributions

P.V., R.J.D.M, and Z.V. designed the experiment; P.V., R.J.D.M, and P.H. collected the imaging data and performed the data pre-processing; P.V. and R.J.D.M developed the convolutional neural network and the transfer learning protocols and performed the statistical analysis; all authors contributed to the interpretation of the results; P.V. and R.J.D.M. wrote the manuscript with the assistance of Z.V. and P.H.

Acknowledgements

We thank Petra Madurka and Annamária Manga for their assistance with data collection. We are very grateful to Tibor Auer for his assistance in data pre-processing.

References

1. LeCun Y, Bengio Y, Hinton G. Deep learning. Nature. 2015;521:nature14539.

2. Cohen JD, Daw N, Engelhardt B, Hasson U, Li K, Niv Y, et al. Computational approaches to fMRI analysis. Nat Neurosci. 2017;20:nn.4499.

3. Vieira S, Pinaya WHL, Mechelli A. Using deep learning to investigate the neuroimaging correlates of psychiatric and neurological disorders: Methods and applications. Neurosci Biobehav Rev. 2017;74:58–75.

4. Meszlényi RJ, Buza K, Vidnyánszky Z. Resting State fMRI Functional Connectivity-Based Classification Using a Convolutional Neural Network Architecture. Front Neuroinformatics [Internet]. 2017 [cited 2017 Nov 13];11. Available from: https://www.frontiersin.org/articles/10.3389/fninf.2017.00061/full

5. Marblestone AH, Wayne G, Kording KP. Toward an Integration of Deep Learning and Neuroscience. Front Comput Neurosci [Internet]. 2016 [cited 2017 Nov 20];10. Available from: https://www.ncbi.nlm.nih.gov/pmc/articles/PMC5021692/

6. Goodfellow I, Bengio Y, Courville A. Deep learning. MIT press; 2016.

7. Krizhevsky A, Sutskever I, Hinton GE. ImageNet Classification with Deep Convolutional Neural Networks. In: Pereira F, Burges CJC, Bottou L, Weinberger KQ, editors. Adv Neural Inf Process Syst 25 [Internet]. Curran Associates, Inc.; 2012 [cited 2017 Nov 21]. p. 1097–1105. Available from: http://papers.nips.cc/paper/4824-imagenet-classification-with-deep-convolutional-neural-networks.pdf

8. Russakovsky O, Deng J, Su H, Krause J, Satheesh S, Ma S, et al. ImageNet Large Scale Visual Recognition Challenge. Int J Comput Vis. 2015;115:211–52.

9. The ImageNet database. [Internet]. [cited 2018 Jan 22]. Available from: http://image-net.org/

10. Arbabshirani MR, Plis S, Sui J, Calhoun VD. Single subject prediction of brain disorders in neuroimaging: Promises and pitfalls. NeuroImage. 2017;145:137–65.

11. Xia M, He Y. Functional connectomics from a "big data" perspective. NeuroImage. 2017;160:152–67.

12. Lemm S, Blankertz B, Dickhaus T, Müller K-R. Introduction to machine learning for brain imaging. NeuroImage. 2011;56:387–99.

13. Pereira F, Mitchell T, Botvinick M. Machine learning classifiers and fMRI: a tutorial overview. NeuroImage. 2009;45:S199–209.

14. Poldrack RA, Gorgolewski KJ. Making big data open: data sharing in neuroimaging. Nat Neurosci. 2014;17:1510–7.

15. Glover GH, Mueller BA, Turner JA, van Erp TGM, Liu TT, Greve DN, et al. Function biomedical informatics research network recommendations for prospective multicenter functional MRI studies. J Magn Reson Imaging. 2012;36:39–54.

16. Pan SJ, Yang Q. A Survey on Transfer Learning. IEEE Trans Knowl Data Eng. 2010;22:1345–59.

17. Yosinski J, Clune J, Bengio Y, Lipson H. How transferable are features in deep neural networks? In: Ghahramani Z, Welling M, Cortes C, Lawrence ND, Weinberger KQ, editors. Adv Neural Inf Process Syst 27 [Internet]. Curran Associates, Inc.; 2014 [cited 2017 Nov 28]. p. 3320–3328. Available from: http://papers.nips.cc/paper/5347-how-transferable-are-features-in-deep-neural-networks.pdf

18. Oquab M, Bottou L, Laptev I, Sivic J. Learning and Transferring Mid-Level Image Representations using Convolutional Neural Networks. 2014 [cited 2017 Nov 28]. p. 1717–24. Available from: https://www.cv-foundation.org/openaccess/content_cvpr_2014/html/Oquab_Learning_and_Transferring_2014_CVPR_paper.html

19. Donahue J, Jia Y, Vinyals O, Hoffman J, Zhang N, Tzeng E, et al. DeCAF: A Deep Convolutional Activation Feature for Generic Visual Recognition. PMLR [Internet]. 2014 [cited 2017 Nov 28]. p. 647–55. Available from: http://proceedings.mlr.press/v32/donahue14.html

20. Sharif Razavian A, Azizpour H, Sullivan J, Carlsson S. CNN Features Off-the-Shelf: An Astounding Baseline for Recognition. 2014 [cited 2017 Nov 28]. p. 806–13. Available from: https://www.cv-foundation.org/openaccess/content_cvpr_workshops_2014/W15/html/Razavian_CNN_Features_Off-the-Shelf_2014_CVPR_paper.html

21. Zeiler MD, Fergus R. Visualizing and Understanding Convolutional Networks. In: Fleet D, Pajdla T, Schiele B, Tuytelaars T, editors. Comput Vis – ECCV 2014 [Internet]. Cham: Springer International Publishing; 2014 [cited 2017 Nov 28]. p. 818–33. Available from: http://link.springer.com/10.1007/978-3-319-10590-1_53

22. Yosinski J, Clune J, Nguyen A, Fuchs T, Lipson H. Understanding Neural Networks Through Deep Visualization. ArXiv150606579 Cs [Internet]. 2015 [cited 2017 Nov 29]; Available from: http://arxiv.org/abs/1506.06579

23. Cole JH, Franke K. Predicting Age Using Neuroimaging: Innovative Brain Ageing Biomarkers. Trends Neurosci. 2017;40:681–90.

24. Cole JH, Poudel RPK, Tsagkrasoulis D, Caan MWA, Steves C, Spector TD, et al. Predicting brain age with deep learning from raw imaging data results in a reliable and heritable biomarker. NeuroImage. 2017;163:115–24.

25. Zuo X-N, Anderson JS, Bellec P, Birn RM, Biswal BB, Blautzik J, et al. An open science resource for establishing reliability and reproducibility in functional connectomics. Sci Data. 2014;1:sdata201449.

26. Blautzik J, Keeser D, Berman A, Paolini M, Kirsch V, Mueller S, et al. Long-Term Test-Retest Reliability of Resting-State Networks in Healthy Elderly Subjects and Patients with Amnestic Mild Cognitive Impairment. J Alzheimers Dis. 2013;34:741–54.

27. Blautzik J, Vetter C, Peres I, Gutyrchik E, Keeser D, Berman A, et al. Classifying fMRI-derived resting-state connectivity patterns according to their daily rhythmicity. NeuroImage. 2013;71:298–306.

28. Mennes M, Biswal BB, Castellanos FX, Milham MP. Making data sharing work: The FCP/INDI experience. NeuroImage. 2013;82:683–91.

29. Wei D, Zhuang K, Chen Q, Yang W, Liu W, Wang K, et al. Structural and functional MRI from a cross-sectional Southwest University Adult lifespan Dataset (SALD). bioRxiv. 2018;177279.

30. Nooner KB, Colcombe SJ, Tobe RH, Mennes M, Benedict MM, Moreno AL, et al. The NKI-Rockland Sample: A Model for Accelerating the Pace of Discovery Science in Psychiatry. Front Neurosci [Internet]. 2012 [cited 2018 Aug 28];6. Available from: https://www.ncbi.nlm.nih.gov/pmc/articles/PMC3472598/

31. Templates and Atlases included with FSL. [Internet]. [cited 2018 Jan 22]. Available from: https://fsl.fmrib.ox.ac.uk/fsl/fslwiki/Atlases

32. Cheng B, Liu M, Suk H-I, Shen D, Zhang D, Initiative ADN. Multimodal manifold-regularized transfer learning for MCI conversion prediction. Brain Imaging Behav. 2015;9:913–26.

33. Cheng B, Liu M, Shen D, Li Z, Zhang D, Initiative the ADN. Multi-Domain Transfer Learning for Early Diagnosis of Alzheimer’s Disease. Neuroinformatics. 2017;15:115–32.

34. Wachinger C, Reuter M. Domain adaptation for Alzheimer’s disease diagnostics. NeuroImage. 2016;139:470–9.

35. Mensch A, Mairal J, Bzdok D, Thirion B, Varoquaux G. Learning Neural Representations of Human Cognition across Many fMRI Studies. In: Guyon I, Luxburg UV, Bengio S, Wallach H, Fergus R, Vishwanathan S, et al., editors. Adv Neural Inf Process Syst 30 [Internet]. Curran Associates, Inc.; 2017 [cited 2017 Dec 12]. p. 5885–5895. Available from: http://papers.nips.cc/paper/7170-learning-neural-representations-of-human-cognition-across-many-fmri-studies.pdf

36. LMU 1 dataset. [Internet]. [cited 2018 Jan 26]. Available from: http://fcon_1000.projects.nitrc.org/indi/CoRR/html/lmu_1.html

37. LMU 2 dataset. [Internet]. [cited 2018 Jan 26]. Available from: http://fcon_1000.projects.nitrc.org/indi/CoRR/html/lmu_2.html

38. LMU 3 dataset. [Internet]. [cited 2018 Jan 26]. Available from: http://fcon_1000.projects.nitrc.org/indi/CoRR/html/lmu_3.html

39. Southwest University Adult Lifespan Dataset (SALD). [Internet]. [cited 2018 Nov 5]. Available from: http://fcon_1000.projects.nitrc.org/indi/retro/sald.html

40. SPM12 By members & collaborators of the Wellcome Trust Centre for Neuroimaging. [Internet]. [cited 2018 Jan 26]. Available from: http://www.fil.ion.ucl.ac.uk/spm/software/spm12/

41. Weissenbacher A, Kasess C, Gerstl F, Lanzenberger R, Moser E, Windischberger C. Correlations and anticorrelations in resting-state functional connectivity MRI: A quantitative comparison of preprocessing strategies. NeuroImage. 2009;47:1408–16.

42. Cordes D, Haughton VM, Arfanakis K, Carew JD, Turski PA, Moritz CH, et al. Frequencies Contributing to Functional Connectivity in the Cerebral Cortex in “Resting-state” Data. Am J Neuroradiol. 2001;22:1326–33.

43. Setsompop K, Gagoski BA, Polimeni JR, Witzel T, Wedeen VJ, Wald LL. Blipped-controlled aliasing in parallel imaging for simultaneous multislice echo planar imaging with reduced g-factor penalty. Magn Reson Med. 2012;67:1210–24.

44. Cauley SF, Polimeni JR, Bhat H, Wald LL, Setsompop K. Interslice leakage artifact reduction technique for simultaneous multislice acquisitions. Magn Reson Med. 2014;72:93–102.

45. FMRIB Software Library v5.0 Created by the Analysis Group, FMRIB, Oxford, UK. [Internet]. [cited 2018 Jan 26]. Available from: https://fsl.fmrib.ox.ac.uk/fsl/fslwiki

46. Beckmann CF, Smith SM. Probabilistic independent component analysis for functional magnetic resonance imaging. IEEE Trans Med Imaging. 2004;23:137–52.

47. Griffanti L, Douaud G, Bijsterbosch J, Evangelisti S, Alfaro-Almagro F, Glasser MF, et al. Hand classification of fMRI ICA noise components. NeuroImage. 2017;154:188–205.

48. Xu J, Moeller S, Strupp J, Auerbach E, Chen L, Feinberg DA, et al. Highly accelerated whole brain imaging using aligned-blipped-controlled-aliasing multiband EPI. Proc 20th Annu Meet ISMRM. 2012.

49. Enhanced Nathan Kline Institute - Rockland Sample [Internet]. Available from: http://fcon_1000.projects.nitrc.org/indi/enhanced/index.html#

50. Nair V, Hinton GE. Rectified Linear Units Improve Restricted Boltzmann Machines. Proc 27th Int Conf Int Conf Mach Learn [Internet]. USA: Omnipress; 2010. p. 807–814. Available from: http://dl.acm.org/citation.cfm?id=3104322.3104425

51. Bridle JS. Probabilistic Interpretation of Feedforward Classification Network Outputs, with Relationships to Statistical Pattern Recognition. Neurocomputing [Internet]. Springer, Berlin, Heidelberg; 1990 [cited 2017 Nov 13]. p. 227–36. Available from: https://link.springer.com/chapter/10.1007/978-3-642-76153-9_28

52. Srivastava N, Hinton G, Krizhevsky A, Sutskever I, Salakhutdinov R. Dropout: A Simple Way to Prevent Neural Networks from Overfitting. J Mach Learn Res. 2014;15:1929–1958.

53. Wager S, Wang S, Liang PS. Dropout Training as Adaptive Regularization. In: Burges CJC, Bottou L, Welling M, Ghahramani Z, Weinberger KQ, editors. Adv Neural Inf Process Syst 26 [Internet]. Curran Associates, Inc.; 2013. p. 351–359. Available from: http://papers.nips.cc/paper/4882-dropout-training-as-adaptive-regularization.pdf

54. Kingma DP, Ba J. Adam: A Method for Stochastic Optimization. ArXiv14126980 Cs [Internet]. 2014 [cited 2018 Jan 22]; Available from: http://arxiv.org/abs/1412.6980

55. Glorot X, Bengio Y. Understanding the difficulty of training deep feedforward neural networks. PMLR [Internet]. 2010 [cited 2017 Nov 13]. p. 249–56. Available from: http://proceedings.mlr.press/v9/glorot10a.html

56. Salzberg SL. On Comparing Classifiers: Pitfalls to Avoid and a Recommended Approach. Data Min Knowl Discov. 1997;1:317–28.

57. Shirer WR, Ryali S, Rykhlevskaia E, Menon V, Greicius MD. Decoding Subject-Driven Cognitive States with Whole-Brain Connectivity Patterns. Cereb Cortex. 2012;22:158–65.

Figure 1. Schematic illustration of the baseline, transfer learning, and back-transfer conditions. Rectangles represent the weights and bias terms in each layer. The color of the rectangles specifies which dataset the layer was originally trained on (green: in-house dataset, blue: public dataset). Open and closed padlocks indicate whether the weights and bias terms were used for initialization or kept constant, respectively. The color of the input and output signs indicate

which dataset was used for testing (the target dataset). Subscripts in the condition names indicate whether the weights and biases in the respective layers were kept constant (Const), initialized on previously learned values (Init), or learned from scratch (Train) when the CCNN was applied to the target dataset. See the Methods section for details.

Table 1. Performance measures of the baseline and transfer learning conditions for the in-house dataset.

Classification	Conv$_{\text{Train}}$ Full$_{\text{Train}}$	Conv$_{\text{Const}}$ Full$_{\text{Const}}$	Conv$_{\text{Const}}$ Full$_{\text{Train}}$	Conv$_{\text{Init}}$ Full$_{\text{Init}}$	Conv$_{\text{Init}}$ Full$_{\text{Train}}$	Conv$_{\text{Const}}$ Full$_{\text{Init}}$	Back-transfer
Accuracy (%)	84.2	86.0	91.2	93.0	93.0	91.2	60.9
AUC	0.919	0.959	0.931	0.945	0.950	0.931	0.720

Figure 2. Network occlusion test results for the $Conv_{Const}Full_{Const}$ and $Conv_{Train}Full_{Train}$ conditions performed on the in-house dataset. The percentage of correctly classified exemplars in the in-house dataset (horizontal axes) are plotted for each occluded functional network (vertical axes). Black dashed lines show the classification accuracies in the corresponding conditions when no network was occluded. Red dashed lines show the accuracy level corresponding to random classification.

Figure 3. Network occlusion test results for the $Conv_{Init}Full_{Init}$ and $Conv_{Init}Full_{Train}$ conditions performed on the in-house dataset. The percentage of correctly classified exemplars in the in-house dataset (horizontal axes) are plotted for each occluded functional network (vertical axes). Black dashed lines show the classification accuracies in the corresponding conditions when no network was occluded. Red dashed lines show the accuracy level corresponding to random classification.

Figure 4. Functional brain networks that strongly contributed to classification by age group performed on the in-house dataset in the baseline and transfer learning conditions. These networks were defined by mapping the constituent functional ROIs identified by Shirer et al. [57] onto the anatomical ROIs in the Harvard-Oxford Atlas. The importance of each network was assessed by occluding the connectivity fingerprints of the constituent ROIs in the input correlation matrices and examining the resulting change in performance when the network classified the in-house exemplars using the weights and biases established in the $Conv_{Const}Full_{Const}$, $Conv_{Train}Full_{Train}$, $Conv_{Init}Full_{Init}$, and $Conv_{Init}Full_{Train}$ conditions. The most important networks were considered to be the ones the occlusion of which resulted in the greatest drop in classification accuracy in the above conditions.

Figure 5. Chronological age regression performance with (yellow) and without (orange) transfer learning. Histograms show the distribution of errors in predicted chronological age in years for the in-house exemplars (vertical axis represents the value of prediction error; horizontal axis represents frequency).

Figure 1.

Click here to access/download;Figure;Figure_1.pdf

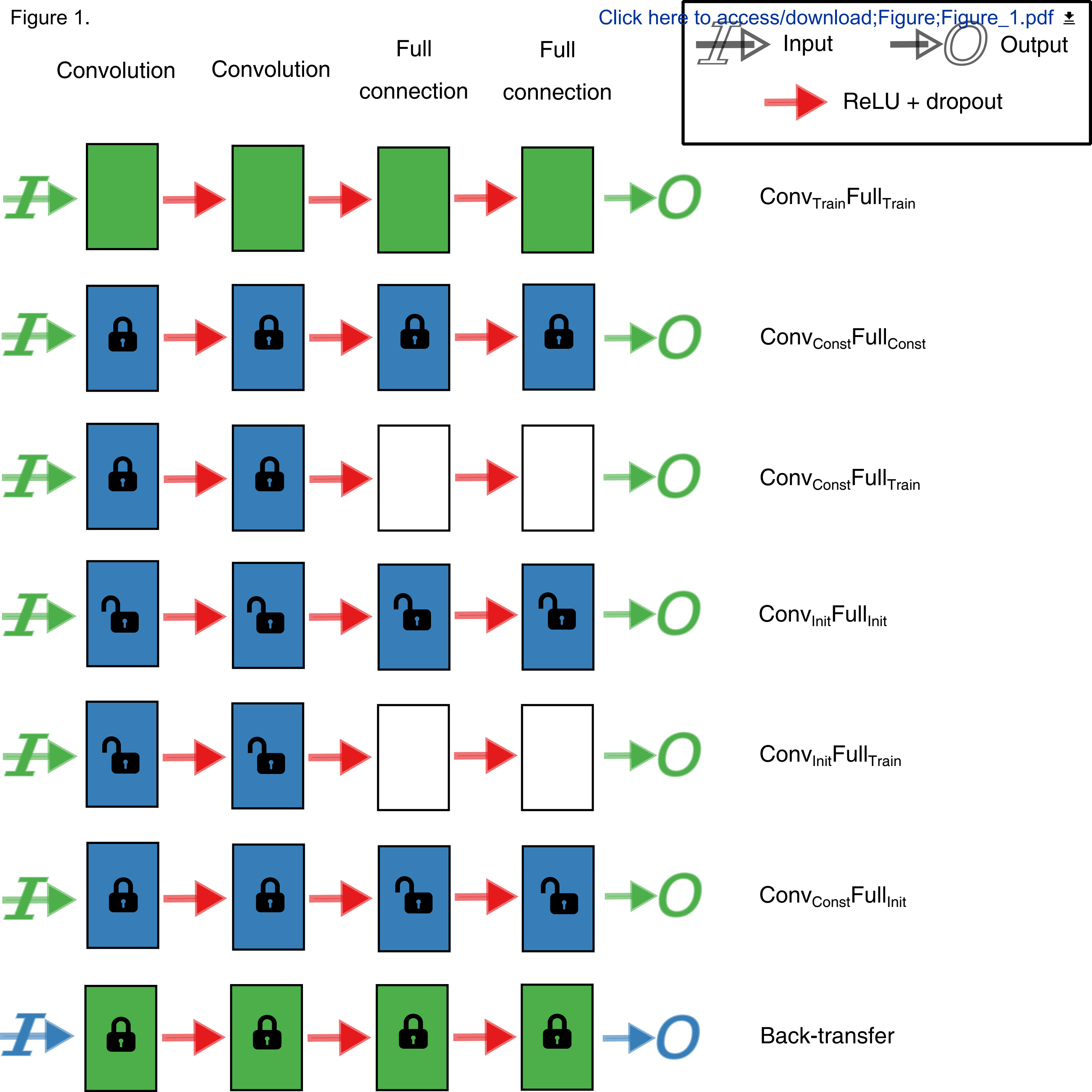

Figure 2.

Click here to access/download;Figure;Figure_2.pdf

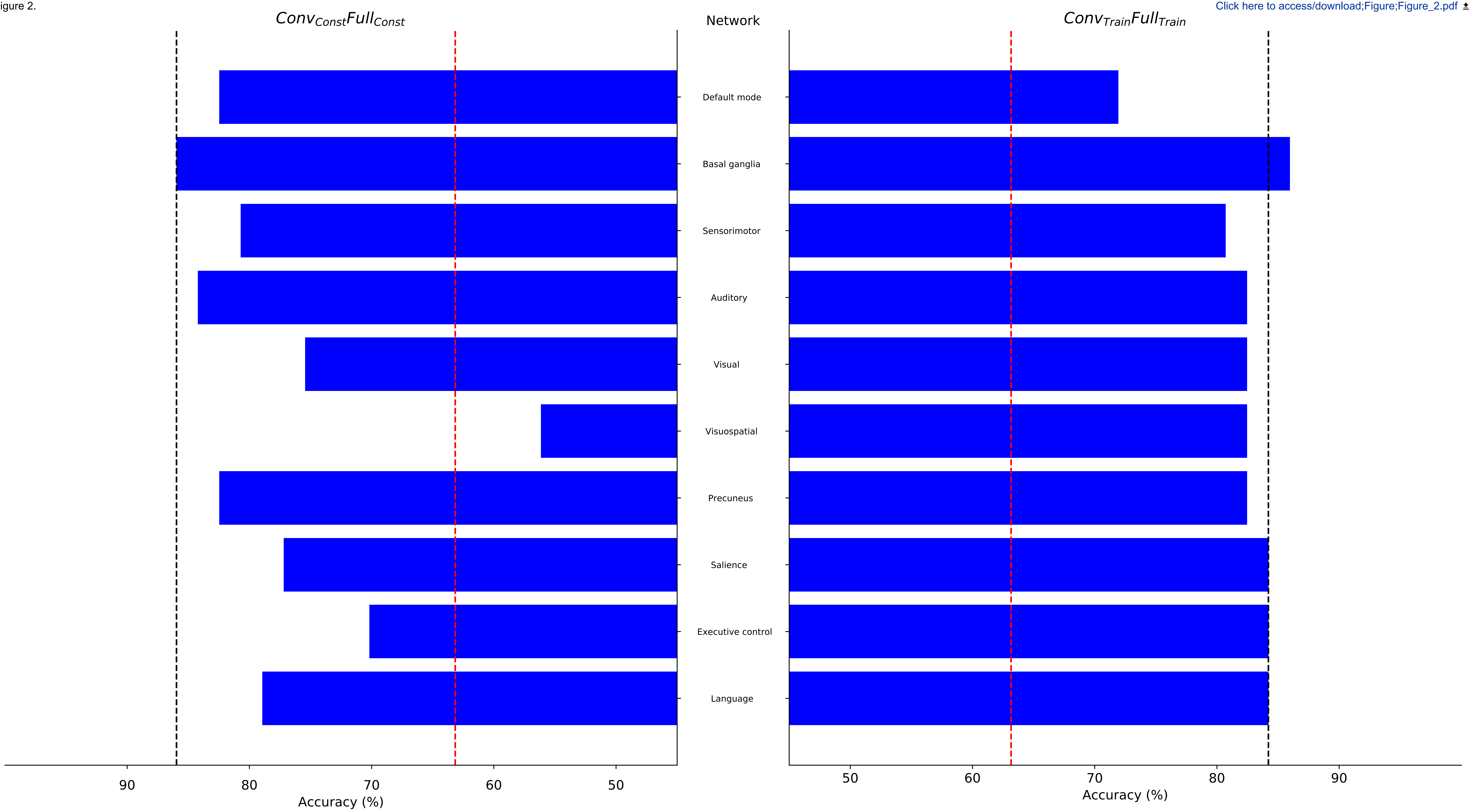

Figure 3.

Click here to access/download;Figure;Figure_3.pdf

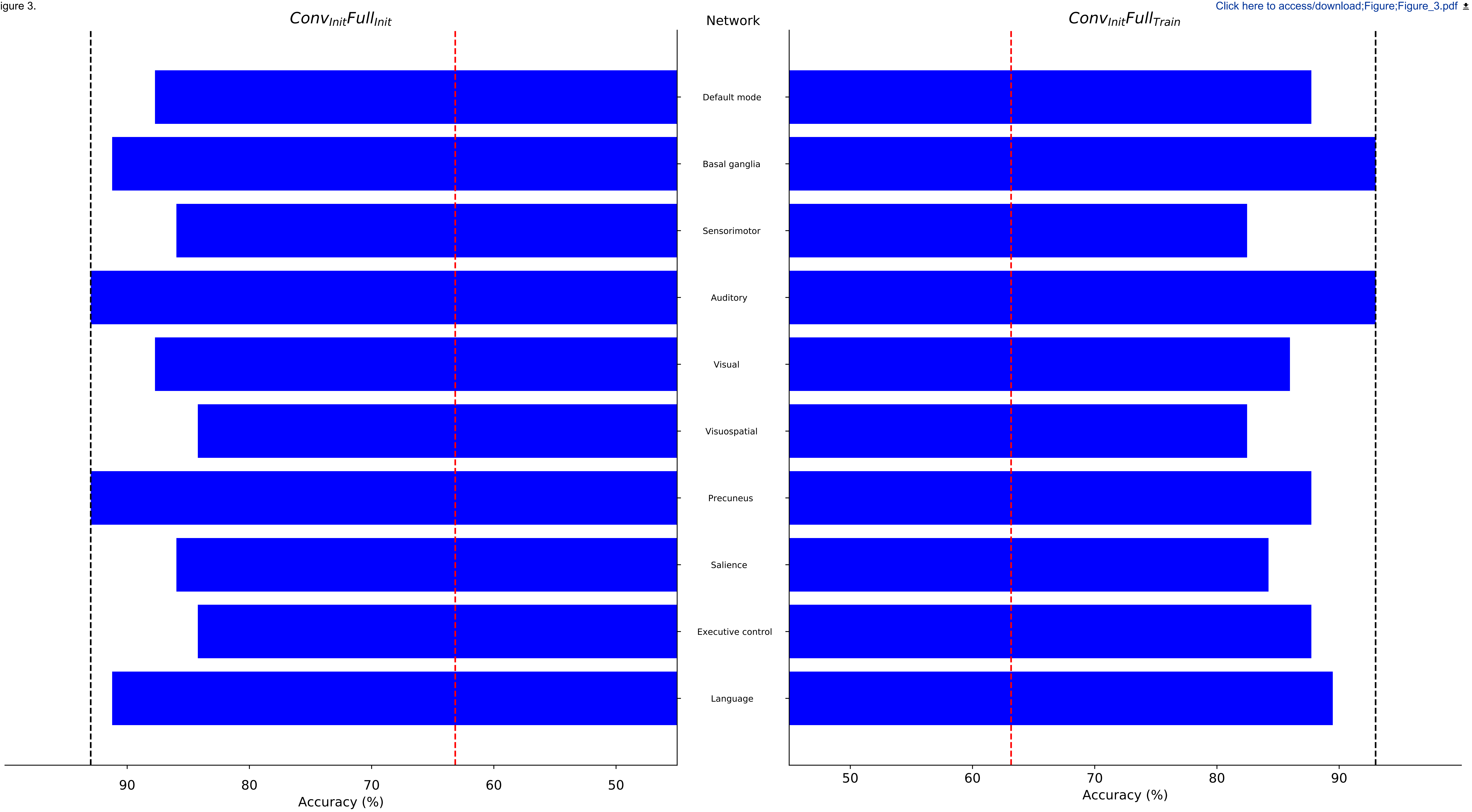

Figure 4.

Click here to access/download;Figure;Figure_4.png

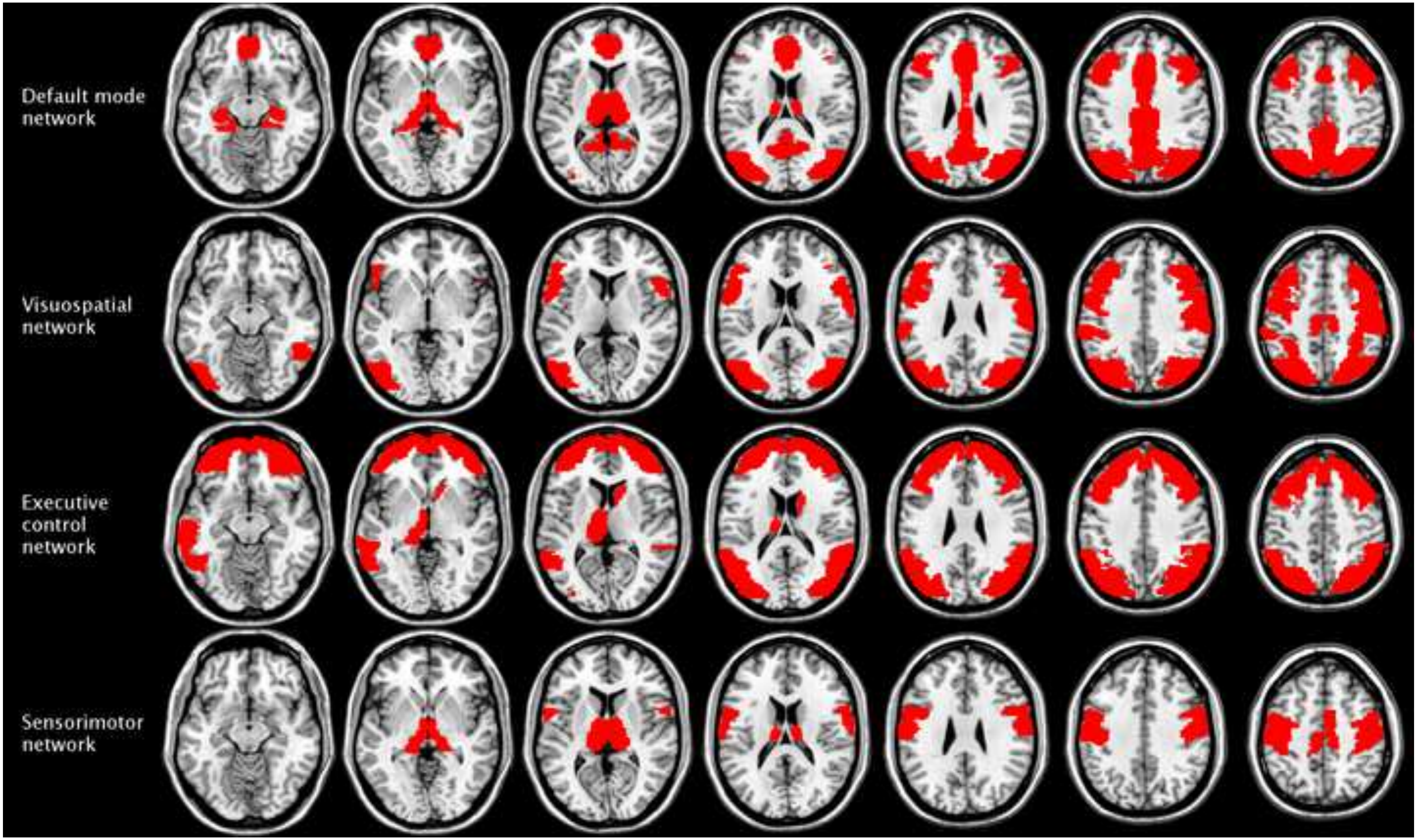

Figure 5.

Click here to access/download;Figure;Figure_5.pdf

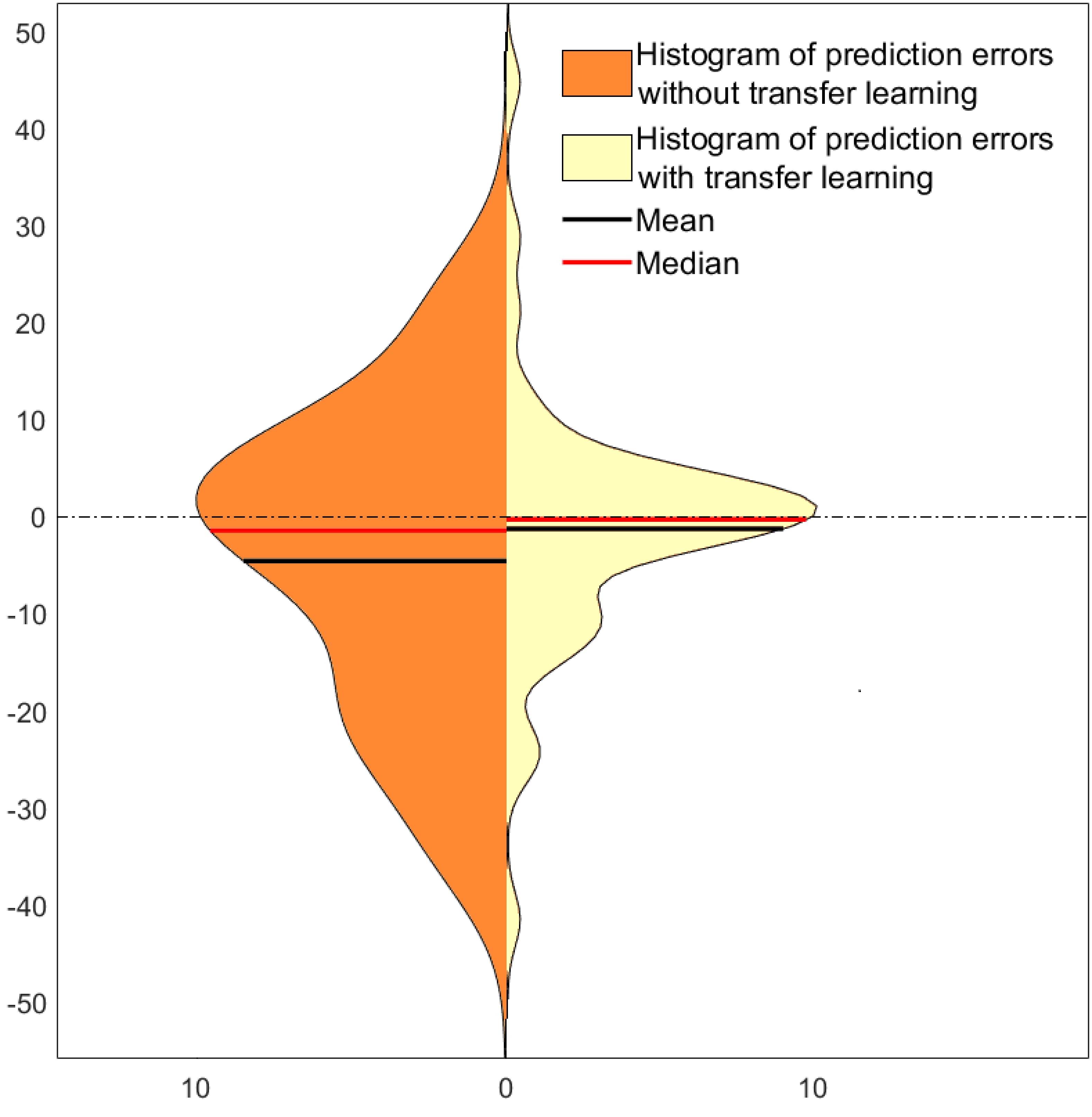

Additional file 1.

Click here to access/download
Supplementary Material
Additional_file_1.pdf

Additional file 2.

Click here to access/download
Supplementary Material
Additional_file_2.pdf

Additional file 3.

Click here to access/download
Supplementary Material
Additional_file_3.pdf

Additional file 4.

Click here to access/download
Supplementary Material
Additional_file_4.pdf

Additional file 5.

Click here to access/download
Supplementary Material
Additional_file_5.pdf

Additional file 6.

Click here to access/download
Supplementary Material
Additional_file_6.pdf

Cover letter

Click here to access/download
Supplementary Material
Cover_letter.pdf

Click here to access/download
Supplementary Material
Response_to_Reviewers.pdf
