# Supplementary material for: Transfer learning improves resting-state functional connectivity pattern analysis using convolutional neural networks
Source: Gigascience. 2018 Nov 5;7(12):giy130. doi: 10.1093/gigascience/giy130 (PMC6283213; doi:10.1093/gigascience/giy130)
Supplement: Supplemental Files [file giy130_supplemental_files.zip › Additional_file_1.pdf]

List of ROIs

1. Left Frontal Pole
2. Right Frontal Pole
3. Left Insular Cortex
4. Right Insular Cortex
5. Left Superior Frontal Gyrus
6. Right Superior Frontal Gyrus
7. Left Middle Frontal Gyrus
8. Right Middle Frontal Gyrus
9. Left Inferior Frontal Gyrus, pars triangularis
10. Right Inferior Frontal Gyrus, pars triangularis
11. Left Inferior Frontal Gyrus, pars opercularis
12. Right Inferior Frontal Gyrus, pars opercularis
13. Left Precentral Gyrus
14. Right Precentral Gyrus
15. Left Temporal Pole
16. Right Temporal Pole
17. Left Superior Temporal Gyrus, anterior division
18. Right Superior Temporal Gyrus, anterior division
19. Left Superior Temporal Gyrus, posterior division
20. Right Superior Temporal Gyrus, posterior division
21. Left Middle Temporal Gyrus, anterior division
22. Right Middle Temporal Gyrus, anterior division
23. Left Middle Temporal Gyrus, posterior division
24. Right Middle Temporal Gyrus, posterior division
25. Left Middle Temporal Gyrus, temporooccipital part
26. Right Middle Temporal Gyrus, temporooccipital part
27. Left Inferior Temporal Gyrus, anterior division
28. Right Inferior Temporal Gyrus, anterior division
29. Left Inferior Temporal Gyrus, posterior division
30. Right Inferior Temporal Gyrus, posterior division
31. Left Inferior Temporal Gyrus, temporooccipital part
32. Right Inferior Temporal Gyrus, temporooccipital part
33. Left Postcentral Gyrus
34. Right Postcentral Gyrus
35. Left Superior Parietal Lobule
36. Right Superior Parietal Lobule
37. Left Supramarginal Gyrus, anterior division
38. Right Supramarginal Gyrus, anterior division
39. Left Supramarginal Gyrus, posterior division
40. Right Supramarginal Gyrus, posterior division
41. Left Angular Gyrus
42. Right Angular Gyrus
43. Left Lateral Occipital Cortex, superior division
44. Right Lateral Occipital Cortex, superior division
45. Left Lateral Occipital Cortex, inferior division
46. Right Lateral Occipital Cortex, inferior division
47. Left Intracalcarine Cortex
48. Right Intracalcarine Cortex
49. Left Frontal Medial Cortex
50. Right Frontal Medial Cortex
51. Left Juxtapositional Lobule Cortex (formerly Supplementary Motor Cortex)
52. Right Juxtapositional Lobule Cortex (formerly Supplementary Motor Cortex)
53. Left Subcallosal Cortex
54. Right Subcallosal Cortex
55. Left Paracingulate Gyrus
56. Right Paracingulate Gyrus
57. Left Cingulate Gyrus, anterior division

58. Right Cingulate Gyrus, anterior division
59. Left Cingulate Gyrus, posterior division
60. Right Cingulate Gyrus, posterior division
61. Left Precuneous Cortex
62. Right Precuneous Cortex
63. Left Cuneal Cortex
64. Right Cuneal Cortex
65. Left Frontal Orbital Cortex
66. Right Frontal Orbital Cortex
67. Left Parahippocampal Gyrus, anterior division
68. Right Parahippocampal Gyrus, anterior division
69. Left Parahippocampal Gyrus, posterior division
70. Right Parahippocampal Gyrus, posterior division
71. Left Lingual Gyrus
72. Right Lingual Gyrus
73. Left Temporal Fusiform Cortex, anterior division
74. Right Temporal Fusiform Cortex, anterior division
75. Left Temporal Fusiform Cortex, posterior division
76. Right Temporal Fusiform Cortex, posterior division
77. Left Temporal Occipital Fusiform Cortex
78. Right Temporal Occipital Fusiform Cortex
79. Left Occipital Fusiform Gyrus
80. Right Occipital Fusiform Gyrus
81. Left Frontal Operculum Cortex
82. Right Frontal Operculum Cortex
83. Left Central Opercular Cortex
84. Right Central Opercular Cortex
85. Left Parietal Operculum Cortex
86. Right Parietal Operculum Cortex
87. Left Planum Polare
88. Right Planum Polare
89. Left Heschl's Gyrus (includes H1 and H2)
90. Right Heschl's Gyrus (includes H1 and H2)
91. Left Planum Temporale
92. Right Planum Temporale
93. Left Supracalcarine Cortex
94. Right Supracalcarine Cortex
95. Left Occipital Pole
96. Right Occipital Pole
97. Left Thalamus
98. Left Caudate
99. Left Putamen
100. Left Pallidum
101. Brain-Stem
102. Left Hippocampus
103. Left Amygdala
104. Left Accumbens
105. Right Thalamus
106. Right Caudate
107. Right Putamen
108. Right Pallidum
109. Right Hippocampus
110. Right Amygdala
111. Right Accumbens
