# Supplementary material for: Transfer learning improves resting-state functional connectivity pattern analysis using convolutional neural networks
Source: Gigascience. 2018 Nov 5;7(12):giy130. doi: 10.1093/gigascience/giy130 (PMC6283213; doi:10.1093/gigascience/giy130)
Supplement: Supplemental Files [file giy130_supplemental_files.zip › Additional_file_4.pdf]

Network	Functional ROIs defined by Shirer et al.	Anatomical ROIs in the Harvard-Oxford Atlas
**Dorsal default mode network**	Medial Prefrontal Cortex, Anterior Cingulate Cortex, Orbitofrontal Cortex	Left Paracingulate Gyrus; Right Paracingulate Gyrus; Left Cingulate Gyrus, anterior division; Right Cingulate Gyrus, anterior division;
	Left Angular Gyrus	Left Lateral Occipital Cortex, superior division
	Right Superior Frontal Gyrus	-
	Posterior Cingulate Cortex, Precuneus	Left and Right Cingulate Gyrus, posterior division; Left and Right Precuneous Cortex
	Midcingulate Cortex	Left & Right Cingulate Gyrus, posterior division
	Right Angular Gyrus	Right Lateral Occipital Cortex, superior division
	Left and Right Thalamus	Left and Right Thalamus
	Left Hippocampus	Left Hippocampus
	Right Hippocampus	Right Hippocampus
**Ventral default mode network**	Left Retrosplenial Cortex, Posterior Cingulate Cortex	Left Precuneous Cortex
	Left Middle Frontal Gyrus	Left Middle Frontal Gyrus
	Left Parahippocampal Gyrus	Left Parahippocampal Gyrus, posterior division;
	Left Middle Occipital Gyrus	Left Lateral Occipital Cortex, superior division
	Right Retrosplenial Cortex, Posterior Cingulate Cortex	Right Cingulate Gyrus, posterior division; Right Precuneous Cortex
	Precuneus	Left and Right Precuneous Cortex
	Right Superior Frontal Gyrus, Middle Frontal Gyrus	Right Middle Frontal Gyrus
	Right Parahippocampal Gyrus	Right Parahippocampal Gyrus, posterior division
	Right Angular Gyrus, Middle Occipital Gyrus	Right Lateral Occipital Cortex, superior division
	Right Lobule IX	-
**Basal ganglia network**	Left Thalamus, Caudate	Left Thalamus, Left Caudate
	Right Thalamus, Caudate, Putamen	Right Thalamus, Right Caudate, Right Putamen
	Left Inferior Frontal Gyrus	Left Inferior Frontal Gyrus, pars opercularis
	Right Inferior Frontal Gyrus	Right Inferior Frontal Gyrus, pars triangularis
	Pons	Brain-Stem
**Sensorimotor network**	Left Precentral Gyrus, Postcentral Gyrus	Left Precentral Gyrus; Left Postcentral Gyrus
	Right Precentral Gyrus, Postcentral Gyrus	Right Precentral Gyrus; Right Postcentral Gyrus
	Right Supplementary Motor Area	Right Precentral Gyrus; Right Juxtapositional Lobule Cortex (formerly Supplementary Motor Cortex)
	Left Thalamus	Left Thalamus
	Bilateral Lobule IV, Lobule V, Lobule VI	-
	Right Thalamus	Right Thalamus

Network	Functional ROIs defined by Shirer et al.	Anatomical ROIs in the Harvard-Oxford Atlas
**Sensorimotor network**	Left Precentral Gyrus, Postcentral Gyrus	Left Precentral Gyrus; Left Postcentral Gyrus
	Right Precentral Gyrus, Postcentral Gyrus	Right Precentral Gyrus; Right Postcentral Gyrus
	Right Supplementary Motor Area	Right Precentral Gyrus; Right Juxtapositional Lobule Cortex (formerly Supplementary Motor Cortex)
	Left Thalamus	Left Thalamus
	Bilateral Lobule IV, Lobule V, Lobule VI	-
	Right Thalamus	Right Thalamus
**Auditory network**	Left Superior Temporal Gyrus, Heschl's Gyrus	Left Central Opercular Cortex; Left Planum Polare; Left Heschl's Gyrus (includes H1 and H2); Left Planum Temporale
	Right Superior Temporal Gyrus	Right Central Opercular Cortex; Right Planum Polare; Right Planum Temporale
	Right Thalamus	Right Thalamus
**Primary visual network**	Calcarine Sulcus	Left and Right Intracalcarine Cortex; Left and Right Supracalcarine Cortex
	Left Thalamus	Left Thalamus
**Higher visual network**	Left Middle Occipital Gyrus, Superior Occipital Gyrus	Left Lateral Occipital Cortex, inferior division; Left Occipital Fusiform Gyrus
	Right Middle Occipital Gyrus, Superior Occipital Gyrus	Right Lateral Occipital Cortex, inferior division; Right Occipital Fusiform Gyrus; Right Occipital Pole
**Visuospatial network**	Left Middle Frontal Gyrus, Superior Frontal Gyrus, Precentral Gyrus	Left Middle Frontal Gyrus
	Left Inferior Parietal Sulcus	Left Superior Parietal Lobule; Left Supramarginal Gyrus, anterior division; Left Lateral Occipital Cortex, superior division
	Left Frontal Operculum, Inferior Frontal Gyrus	Left Inferior Frontal Gyrus, pars triangularis; Left Inferior Frontal Gyrus, pars opercularis; Left Precentral Gyrus
	Left Inferior Temporal Gyrus	Left Lateral Occipital Cortex, inferior division
	Right Middle Frontal Gyrus	Right Middle Frontal Gyrus
	Right Inferior Parietal Lobule	Right Postcentral Gyrus; Right Superior Parietal Lobule; Right Supramarginal Gyrus, anterior division; Right Lateral Occipital Cortex, superior division
	Right Frontal Operculum, Inferior Frontal Gyrus	Right Inferior Frontal Gyrus, pars opercularis; Right Precentral Gyrus
	Right Middle Temporal Gyrus	Right Inferior Temporal Gyrus, temporooccipital part
	Left Lobule VIII, Lobule VIIb	-
	Right Lobule VIII, Lobule VIIb	-
	Right Lobule VI, Crus I	-

Network	Functional ROIs defined by Shirer et al.	Anatomical ROIs in the Harvard-Oxford Atlas
**Precuneus network**	Midcingulate Cortex, Posterior Cingulate Cortex	Left and Right Cingulate Gyrus, posterior division
	Precuneus	Left and Right Precuneous Cortex
	Left Angular Gyrus	Left Angular Gyrus; Left Lateral Occipital Cortex, superior division
	Right Angular Gyrus	Right Angular Gyrus; Right Lateral Occipital Cortex, superior division
**Anterior salience Network**	Left Middle Frontal Gyrus	Left Frontal Pole
	Left Insula	Left Insular Cortex
	Anterior Cingulate Cortex, Medial Prefrontal Cortex, Supplementary Motor Area	Left and Right Paracingulate Gyrus
	Right Middle Frontal Gyrus	Right Frontal Pole
	Right Insula	Right Insular Cortex
	Left Lobule VI, Crus I	-
	Right Lobule VI, Crus I	-
**Posterior salience network**	Left Middle Frontal Gyrus	Left Middle Frontal Gyrus
	Left Supramarginal Gyrus, Inferior Parietal Gyrus	Left Supramarginal Gyrus, anterior and posterior division
	Left Precuneus	Left Precuneous Cortex
	Right Midcingulate Cortex	Right Precentral Gyrus
	Right Superior Parietal Gyrus, Precuneus	Right Superior Parietal Lobule
	Right Supramarginal Gyrus, Inferior Parietal Gyrus	Right Supramarginal Gyrus, anterior and posterior division
	Left Thalamus	Left Thalamus
	Lobule VI	-
	Left Posterior Insula, Putamen	Left Insular Cortex
	Right Thalamus	Right Thalamus
	Lobule VI	-
	Right Posterior Insula	Right Insular Cortex

Network	Functional ROIs defined by Shirer et al.	Anatomical ROIs in the Harvard-Oxford Atlas
**Left executive control network**	Left Middle Frontal Gyrus, Superior Frontal Gyrus	Left Superior Frontal Gyrus; Left Middle Frontal Gyrus
	Left Inferior Frontal Gyrus, Orbitofrontal Gyrus	Left Frontal Pole
	Left Superior Parietal Gyrus, Inferior Parietal Gyrus, Precuneus, Angular Gyrus	Left Supramarginal Gyrus, posterior division; Left Angular Gyrus; Left Lateral Occipital Cortex, superior division
	Left Inferior Temporal Gyrus, Middle Temporal Gyrus	Left Middle Temporal Gyrus, posterior division; Left Middle Temporal Gyrus, temporooccipital part; Left Inferior Temporal Gyrus, temporooccipital part
	Right Crus I	-
	Left Thalamus	Left Thalamus
**Right executive control network**	Right Middle Frontal Gyrus, Right Superior Frontal Gyrus	Right Frontal Pole; Right Superior Frontal Gyrus; Right Middle Frontal Gyrus
	Right Middle Frontal Gyrus	Right Frontal Pole
	Right Inferior Parietal Gyrus, Supramarginal Gyrus, Angular Gyrus	Right Supramarginal Gyrus, posterior division; Right Angular Gyrus; Right Lateral Occipital Cortex, superior division
	Right Superior Frontal Gyrus	Right Superior Frontal Gyrus
	Left Crus I, Left Crus II, Lobule VI	-
	Right Caudate	Right Caudate
**Language network**	Inferior Frontal Gyrus	Left Inferior Frontal Gyrus, pars triangularis; Left Inferior Frontal Gyrus, pars opercularis
	Left Middle Temporal Gyrus	Left Middle Temporal Gyrus, anterior division
	Left Middle Temporal Gyrus, Angular Gyrus	Left Middle Temporal Gyrus, posterior division
	Middle Temporal Gyrus, Superior Temporal Gyrus, Supramarginal Gyrus, Angural Gyrus	Left Supramarginal Gyrus, posterior division; Left Angular Gyrus; Left Lateral Occipital Cortex, superior division
	Right Inferior Frontal Gyrus	Right Frontal Orbital Cortex
	Right Supramarginal Gyrus, Superior Temporal Gyrus, Middle Temporal Gyrus	Right Middle Temporal Gyrus, posterior division; Right Middle Temporal Gyrus, temporooccipital part; Right Angular Gyrus
	Left Crus I	-
