# Supplementary material for: Transfer learning improves resting-state functional connectivity pattern analysis using convolutional neural networks
Source: Gigascience. 2018 Nov 5;7(12):giy130. doi: 10.1093/gigascience/giy130 (PMC6283213; doi:10.1093/gigascience/giy130)
Supplement: Supplemental Files [file giy130_supplemental_files.zip › Additional_file_5.pdf]

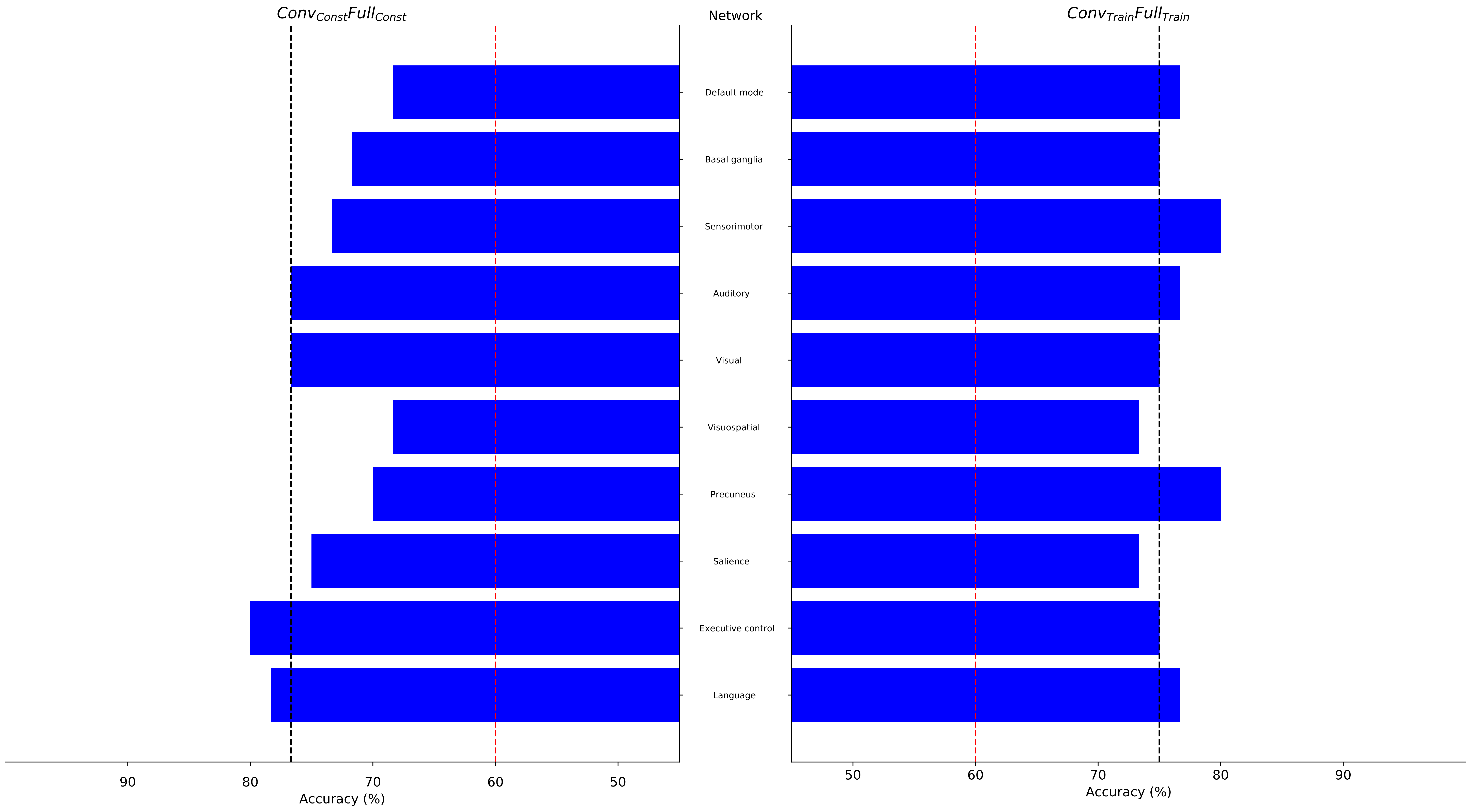

$Conv_{Const}Full_{Const}$
Network
$Conv_{Train}Full_{Train}$
Default mode
Basal ganglia
Sensorimotor
Auditory
Visual
Visuospatial
Precuneus
Salience
Executive control
Language
90
80
70
60
50
Accuracy (%)
50
60
70
80
90
Accuracy (%)
