# Supplementary figures and images for: Transfer learning improves resting-state functional connectivity pattern analysis using convolutional neural networks
Source: Gigascience. 2018 Nov 5;7(12):giy130. doi: 10.1093/gigascience/giy130 (PMC6283213; doi:10.1093/gigascience/giy130)

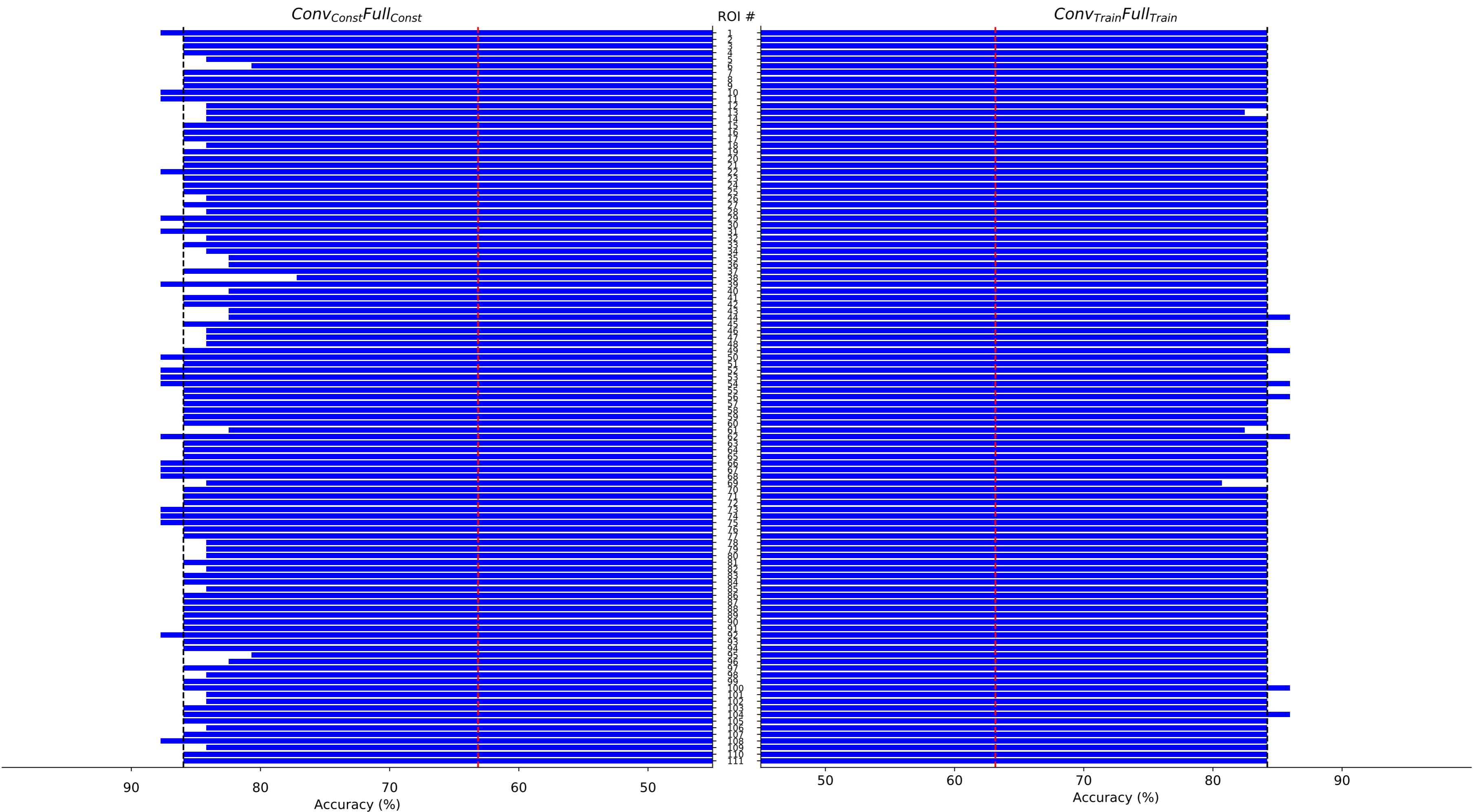

Supplement: Supplemental Files [file giy130_supplemental_files.zip › Additional_file_2.pdf]

*Conv<sub>Init</sub>Full<sub>Init</sub>*

ROI #

*Conv<sub>Init</sub>Full<sub>Train</sub>*

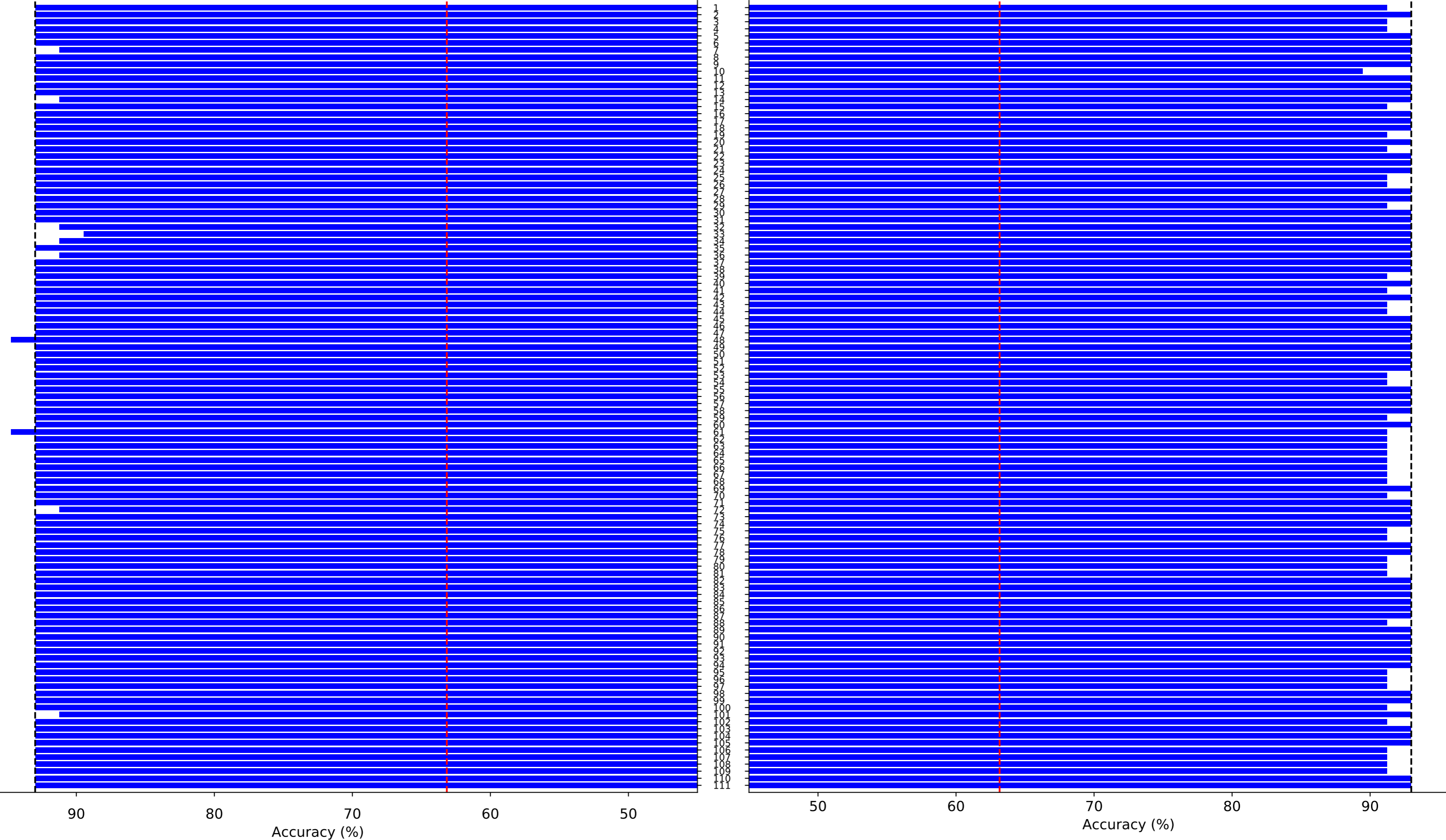

Supplement: Supplemental Files [file giy130_supplemental_files.zip › Additional_file_3.pdf]

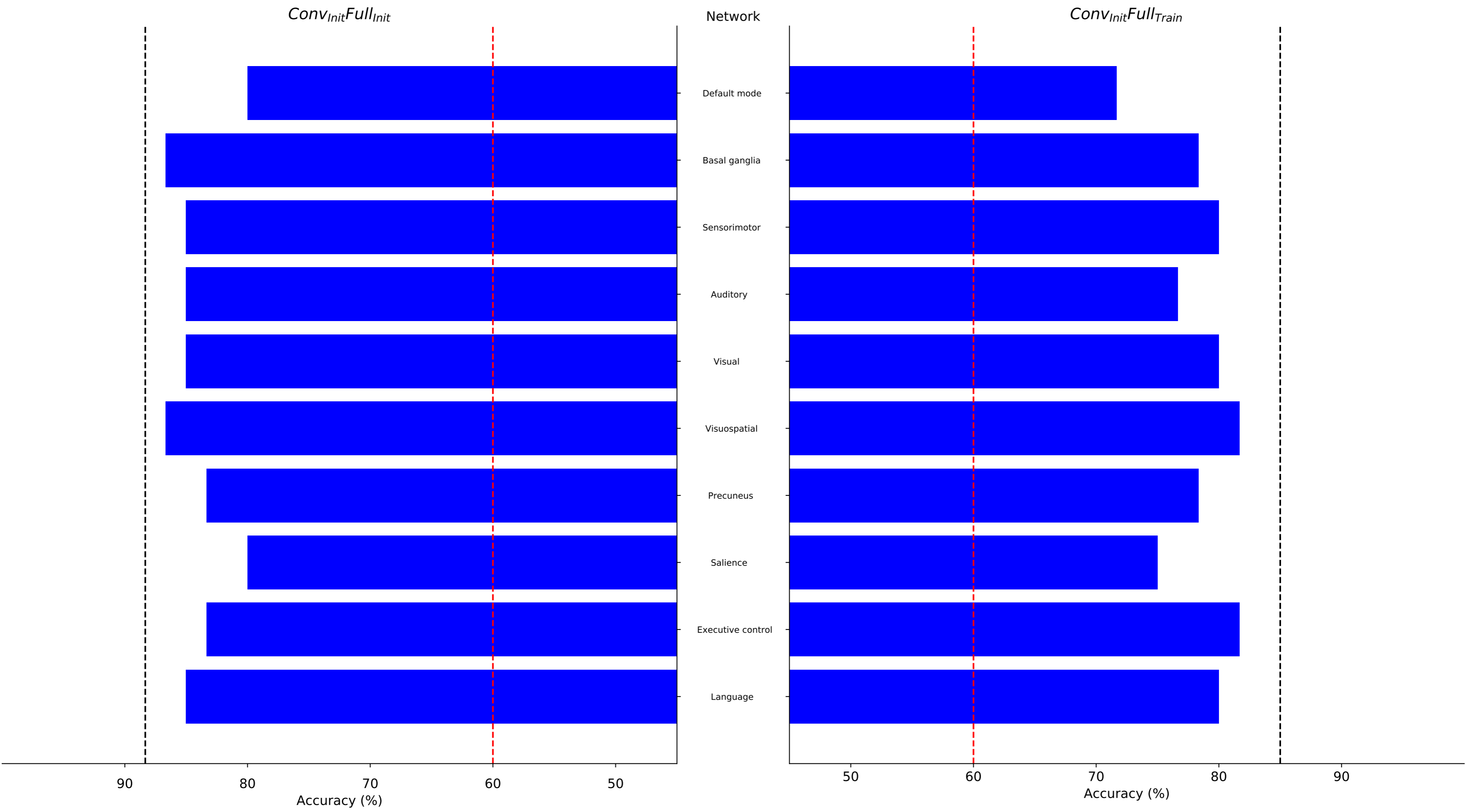

Supplement: Supplemental Files [file giy130_supplemental_files.zip › Additional_file_6.pdf]
