# Supplementary material for: Transfer learning improves resting-state functional connectivity pattern analysis using convolutional neural networks
Source: Gigascience. 2018 Nov 5;7(12):giy130. doi: 10.1093/gigascience/giy130 (PMC6283213; doi:10.1093/gigascience/giy130)
Supplement: Response_to_Reviewer_Comments_Original_Submission.pdf [file giy130_response_to_reviewer_comments_original_submission.pdf]

Author's Response To Reviewer Comments

Close

We are very grateful to the Editor and the Reviewers for their comments and suggestions that helped us improve the quality of the manuscript. Our detailed responses, along with the cover letter, can be found at the end of the manuscript after the supplementary material. We felt it was necessary to use the PDF format because we included several figures and a formula as well in the response.

Close
