# Supplementary material for: Transfer learning improves resting-state functional connectivity pattern analysis using convolutional neural networks
Source: Gigascience. 2018 Nov 5;7(12):giy130. doi: 10.1093/gigascience/giy130 (PMC6283213; doi:10.1093/gigascience/giy130)
Supplement: Response_to_Reviewer_Comments_Revision_1.pdf [file giy130_response_to_reviewer_comments_revision_1.pdf]

Author's Response To Reviewer Comments

Close

Please see our responses to the comments attached in a separate file.

Close
