# Supplementary material for: Transfer learning improves resting-state functional connectivity pattern analysis using convolutional neural networks
Source: Gigascience. 2018 Nov 5;7(12):giy130. doi: 10.1093/gigascience/giy130 (PMC6283213; doi:10.1093/gigascience/giy130)
Supplement: Response_to_Reviewer_Comments_Revision_2.pdf [file giy130_response_to_reviewer_comments_revision_2.pdf]

Author's Response To Reviewer Comments

Close

We are very grateful to the Reviewer and the Editor for thoroughly examining the manuscript. We have now included the ORCID IDs of the authors in the manuscript and changed the author contribution to a tabular format according to the CASRAI CRediT taxonomy. We also registered the software programs implementing the networks as well as the image pre-processing on SciCrunch and included the respective RRIDs in the manuscript.

Close
