# Supplementary material for: Transfer learning improves resting-state functional connectivity pattern analysis using convolutional neural networks
Source: Gigascience. 2018 Nov 5;7(12):giy130. doi: 10.1093/gigascience/giy130 (PMC6283213; doi:10.1093/gigascience/giy130)
Supplement: Reviewer_1_Report_(Original_Submission) -- Alexandre Franco, Ph.D. — 05 Mar 2018 Reviewed [file giy130_reviewer_1_report_(original_submission).pdf]

Reviewer Report

Title: **Transfer learning improves resting-state functional connectivity pattern analysis using convolutional neural networks**

Version: **Original Submission** **Date:** 4/11/2018

Reviewer name: **Alexandre Franco, Ph.D.**

Reviewer Comments to Author:

The paper by Vaki et. al entitled "Transfer learning improves resting-state functional connectivity pattern analysis using convolutional neural networks", shows the applicability of using "transfer learning" of CNN on resting state fMRI data. The group showed that initially training the CNN on a large dataset (i.e. public dataset) and then transferring this network to be used on a smaller dataset (i.e. in-house), there was an improvement in predicting the age of the subject. The paper is focused on the applicability of transfer learning and its potential to be used in neuroimaging studies. This manuscript shows promising results for the use of transfer learning, considering that the data (public and in-house) where acquired on scanners from different manufactures and different T2* protocols were used.

The paper is well written and easy to comprehend. Additionally, the code available on github is well documented.

However, there are some questions that arise when reviewing the manuscript:

Could the use of data from the public dataset in which the subject were scanned more than once impact the training of the CNN? Additionally, there are 41+49=90 subjects (209+146 samples) being used from the public data, and 57 (28 young and 29 old) from the in-house data. From the public data, there is a bias of an average of ~5 visits for the young subjects and average of ~3 visits for the older subjects. Could this at one point impact the results? A confusion matrix showing the classification accuracy for each group would be helpful.

Visualization of Figure 3 could improve greatly if the 3 brain images are combined by showing the overlap of the ROIs. For example, use red (A+B+C), blue (A+B), green (A+C), …
It would make it easier for the reader to interpret the overlap between ROIs.

Preprocessing was different for both datasets.
For example:
1. For the public dataset, whole-brain signal was regressed out the whole-brain signals. This was not performed on the in-house data.
2. ICA was used to remove artifacts on the in-house data. This was not performed on the public dataset.
Could this have impacted the results? Preprocessing has big impact on neuroimaging results, as showed in the manuscript referenced by the authors [43].

Details on the statistics used in the manuscript need to be provided. For example:
Page 8, line 21: How was this p-value calculated?
Page 22, line 5, saying that a paired t-test was performed. The t-test was performed on what metric? The absolute error?

What was the computation time necessary to train each network?

Minor concerns:

In the abstract, state how much improvement there was when using transfer learning (i.e. give a number).

Code used for preprocessing the functional data should be provided.

Limitation of the study:
Data from the "public" dataset was from one site only. This reduces the generalizability of the proposed method.

Methods

Are the methods appropriate to the aims of the study, are they well described, and are necessary controls included? Yes

Conclusions

Are the conclusions adequately supported by the data shown? Yes

Reporting Standards

Does the manuscript adhere to the journal's guidelines on minimum standards of reporting? Yes

Choose an item.

Statistics

Are you able to assess all statistics in the manuscript, including the appropriateness of statistical tests used? Yes, and I have assessed the statistics in my report.

Quality of Written English

Please indicate the quality of language in the manuscript: Acceptable

Declaration of Competing Interests

Please complete a declaration of competing interests, considering the following questions:

- Have you in the past five years received reimbursements, fees, funding, or salary from an organisation that may in any way gain or lose financially from the publication of this manuscript, either now or in the future?
- Do you hold any stocks or shares in an organisation that may in any way gain or lose financially from the publication of this manuscript, either now or in the future?
- Do you hold or are you currently applying for any patents relating to the content of the manuscript?
- Have you received reimbursements, fees, funding, or salary from an organization that holds or has applied for patents relating to the content of the manuscript?
- Do you have any other financial competing interests?

- Do you have any non-financial competing interests in relation to this paper?

If you can answer no to all of the above, write 'I declare that I have no competing interests' below. If your reply is yes to any, please give details below.

I have no conflict of interest

I agree to the open peer review policy of the journal. I understand that my name will be included on my report to the authors and, if the manuscript is accepted for publication, my named report including any attachments I upload will be posted on the website along with the authors' responses. I agree for my report to be made available under an Open Access Creative Commons CC-BY license (http://creativecommons.org/licenses/by/4.0/). I understand that any comments which I do not wish to be included in my named report can be included as confidential comments to the editors, which will not be published.

I agree to the open peer review policy of the journal

To further support our reviewers, we have joined with Publons, where you can gain additional credit to further highlight your hard work (see: https://publons.com/journal/530/gigascience). On publication of this paper, your review will be automatically added to Publons, you can then choose whether or not to claim your Publons credit. I understand this statement.

Yes Choose an item.
