# Supplementary material for: Transfer learning improves resting-state functional connectivity pattern analysis using convolutional neural networks
Source: Gigascience. 2018 Nov 5;7(12):giy130. doi: 10.1093/gigascience/giy130 (PMC6283213; doi:10.1093/gigascience/giy130)
Supplement: Reviewer_2_Report_(Original_Submission) -- Heung-Il Suk — 12/4/2018 Reviewed [file giy130_reviewer_2_report_(original_submission).pdf]

Reviewer Report

Title: **Transfer learning improves resting-state functional connectivity pattern analysis using convolutional neural networks**

Version: **Original Submission** **Date:** 4/12/2018

Reviewer name: **Heung-Il Suk**

Reviewer Comments to Author:

This paper presents a way of using knowledge learned from other large dataset, i.e., transfer learning, for age category classification and prediction. The authors used a CNN with two convolution layers and two fully connected layers for both tasks. While the experimental results show an improvement via transfer learning, there are some concerns that should be considered carefully and justified.

- In order to better justify the validity of transfer learning with the CCNN, it is recommended to further conduct experiments on other public datasets such as ADNI, OASIS, OpenfMRI.

- In a classification task, there is no statistical significance in performance improvement. The reported p-values are 0.966, 0.113, 0.145, 0.063.

- What is the objective of the back-transfer? This work assumes the inapplicability of deep network trained with a limited number of samples.

- This reviewer doesn't agree that the high absolute values in the filters make contributions to performance improvement. It's not justified and there is no proof in either an empirical or a theoretical manner. In page 10, line 3-15, for those statements, there should be more supportive information provided.

- In a regression task, (page 10, line 23), the performance was much lower than those reported in [24].

- It is curious about the classification and/or regression performance over the public dataset, between datasets, via transfer learning.

- Page 13, line 18-21: From a feature representation perspective, it is not reasonable to say that the low-level features whose absolute weights are high are necessarily informative for the high-level tasks. There should be provided the supportive information or data.

- It is not clear how the transfer learning could successfully handle the multi-center or multi-site problem, which is one of the main issues in this work.

- Page 14, line 23-24: This reviewer cannot find the related results that support this statement.

- Regarding the dropout regularization, did the technique apply to both convolution and fully-connected layers? What about the weight decay and momentum? These were also considered for training?

Methods

Are the methods appropriate to the aims of the study, are they well described, and are necessary controls included? Yes

Conclusions

Are the conclusions adequately supported by the data shown? No

Reporting Standards

Does the manuscript adhere to the journal's guidelines on minimum standards of reporting? Yes

Choose an item.

Statistics

Are you able to assess all statistics in the manuscript, including the appropriateness of statistical tests used? No, and I do not feel adequately qualified to assess the statistics.

Quality of Written English

Please indicate the quality of language in the manuscript: Needs some language corrections before being published

Declaration of Competing Interests

Please complete a declaration of competing interests, considering the following questions:

- Have you in the past five years received reimbursements, fees, funding, or salary from an organisation that may in any way gain or lose financially from the publication of this manuscript, either now or in the future?
- Do you hold any stocks or shares in an organisation that may in any way gain or lose financially from the publication of this manuscript, either now or in the future?
- Do you hold or are you currently applying for any patents relating to the content of the manuscript?
- Have you received reimbursements, fees, funding, or salary from an organization that holds or has applied for patents relating to the content of the manuscript?
- Do you have any other financial competing interests?
- Do you have any non-financial competing interests in relation to this paper?

If you can answer no to all of the above, write 'I declare that I have no competing interests' below. If your reply is yes to any, please give details below.

I have no competing interests

I agree to the open peer review policy of the journal. I understand that my name will be included on my report to the authors and, if the manuscript is accepted for publication, my named report including any attachments I upload will be posted on the website along with the authors' responses. I agree for my report to be made available under an Open Access Creative Commons CC-BY license (http://creativecommons.org/licenses/by/4.0/). I understand that any comments which I do not wish to be included in my named report can be included as confidential comments to the editors, which will not be published.

I agree to the open peer review policy of the journal

To further support our reviewers, we have joined with Publons, where you can gain additional credit to further highlight your hard work (see: https://publons.com/journal/530/gigascience). On publication of this paper, your review will be automatically added to Publons, you can then choose whether or not to claim your Publons credit. I understand this statement.

Yes Choose an item.
